# Selection of pallet management strategies from the perspective of supply chain cost with Anylogic software

**DOI:** 10.1371/journal.pone.0217995

**Published:** 2019-06-06

**Authors:** Jianwei Ren, Qingqing Zhao, Bo Liu, Chunhua Chen

**Affiliations:** 1 Transportation Institute, Inner Mongolia University, Hohhot, Inner Mongolia, China; 2 School of Mathematical Sciences, Inner Mongolia University, Hohhot, Inner Mongolia, China; 3 School of Business, University of Plymouth, Plymouth, Devon, United Kingdom; 4 School of Mechanical Engineering, University of Science and Technology, Beijing, Beijing, China; 5 School of Business Administration, Jiangxi University of Finance and Economics, Nanchang, Jiangxi, China; 6 Inner Mongolia Branch of Agricultural Bank of China, Hohhot, Inner Mongolia, China; Shandong University of Science and Technology, CHINA

## Abstract

Pallet is a very important innovation in logistics industry. Pallets are so widely used that we can find them in nearly every logistical operation scenario. In order to manage pallets efficiently, researchers have developed several pallet management strategies (PMS). The most common and widely accepted PMS includes extensive management of pallets (EMP), transfer of pallet’s ownership (TPO), and pallet rent (PR). This paper addresses mainly on how to help pallet managers choose a certain kind of PMS from the perspective of supply chain cost. Firstly, cost models of three kinds of PMS are presented. Secondly, all parameters involved in the models are valued based on data that is collected from industry survey. The results show that the cost of PR is constantly lower than EMP, and also lower than TPO when the operation period is no more than 37 months. Finally, the effect of several important parameters on the cost is studied by sensitivity analysis. The selection strategies of PMS are proposed based on the results.

## Introduction

As an innovation in logistics industry, pallet is known for “Pallets move the world” and “Pallets are the unsung hero of the world of logistics” [1]. Pallets are so widely used that we can find them in nearly every logistical operation scenario, such as packaging, transportation, warehousing, loading and unloading, etc. The use of pallets has brought normalization and standardization to logistics. Pallets are as well regarded instrumental in protecting cargoes and improving logistics efficiency. Pallets can be made of wood, plastics, steel, and so on. The most common material used in pallets is wood [2]. Despite the diversity in the structure of pallets, flat pallet has gained the majority in use. However, there are six dimensions for plat pallet sanctioned by ISO (the International Organization for Standardization), which makes the pallet management become really challenging.

In order to manage pallets efficiently, researchers have developed several pallet management strategies (PMS). The most common and widely accepted PMS includes extensive management of pallets (EMP), transfer of pallet’s ownership (TPO), and pallet rent (PR).

This paper focuses mainly on how to help pallet managers choose a proper kind of PMS from the perspective of supply chain cost based on data collected from filed survey. Firstly, literatures on pallet management are reviewed. Then, three PMS are analyzed. Meanwhile, the supply chain cost models of the three PMS are developed and the costs of the three PMS in different situations are compared by Anylogic software. Finally some conclusions and suggestions are proposed.

## Literature review on pallet management

There are a lot of literatures on pallet management. For instance, the benefit of pallet pooling has been well studied. Auguston, Don, Witt, and Raballand & Carroll stated that pallet pooling can not only save logistics cost, but also improve logistics efficiency [3–6]. Brindley, Zhou et al., and Zhang presented that the development of pallet pooling can reduce the financial and environmental cost [7–9].

Several studies research on pallet supply chain management. Elia and Gnoni designed a closed loop system for pallet management [10]. Zhao proposed a supply chain information service frame model for pallet management based on XML [11]. Li et al. presented a pallet pooling information platform based on cloud computing [12]. Gnoni and Rollo, Kim and Glock, Gnimpieba et al., and Ren et al. focused on how to track pallets with the implementation of tracking devices [13–16].

Some efforts analyze how to allocate pallets. Mosqueda and Brindley pointed out that position was everything [17–18]. Ren et al., Ni et al., Wu et al., and Ren et al. developed pallet allocation models to help managers make decisions with different information background [19–22]. Zhou et al. and Doungpattra et al. studied how to minimize the cost of pallet allocation over railway and pet food industry, respectively [23–25].

The environmental impact of pallet operations is a hot topic. Carrano et al., Bengtssona & Logiea, and Tornese et al. proved that pooled pallets performed more environmentally-friendly than un-pooled pallets [26–29].

There are very few literatures on the selection of PMS from the perspective of a single company. Ray et al. proposed that pallet rental system cost more than pallet purchasing system [30]. Gnoni et al. compared the immediate interchange and postponed interchange from the perspective of cost, supplier’s service time, and internal operations [31]. Roy et al. found that both buy/sell programs and leased pallet pooling programs cost more than single-use expendable pallet approach [32].

Standing on a different view, this paper compares three PMS from the perspective of supply chain instead of a single company, because the competition is no longer between enterprises but between supply chains.

## Problem description

EMP system is nowadays the most popular PMS in the world. The advantage of EMP system is that companies can specify the exact pallet’s size, quality, and material for loading their products. However, in the system of EMP, pallets are normally not traveled with the cargoes, but only used in a certain company. If cargoes are transported from a company (company A) to another one (company B), they have to be unloaded from company A’s pallets and loaded on company B’s pallets afterwards. It actually costs more and ruins logistics efficiency. As the cargoes are loaded and unloaded repeatedly, pallets are more likely to be worn out in EMP. Because most of pallets are wooden pallets and some of them are single-use expendable pallets [32], the EMP is therefore regarded as environmentally-unfriendly.

TPO and PR are two kinds of pallet pooling modes. Pooled pallets can be transported with the cargoes loaded on them. So, pallet pooling can significantly enhance logistics efficiency and decrease logistics cost [33]. Pallet pooling can also reduce the use of pallets, which results in less use of material resources (wood, plastic, steel, etc.) and lower environmental cost. In a pallet pool, pallets usually possess high-quality and standard. Moreover, the dimensions of pallet, experimental methods, and the methods of quality controlling and quality identification are strictly stipulated. The two kinds of pallet management strategies are relatively not so highly adopted in the world in spite of many advantages. There are at least two reasons as follows. (1) In the system of TPO, consignees have to purchase pallets from consignors. In the system of PR, it is not easy to recycle all the pallet assets at the end of use phase, because some pallets may be still in use at certain vertices of a supply chain. (2) The size, quality, and material of pallets may not suit for loading products if companies choose the TPO or PR.

**Fig 1:**
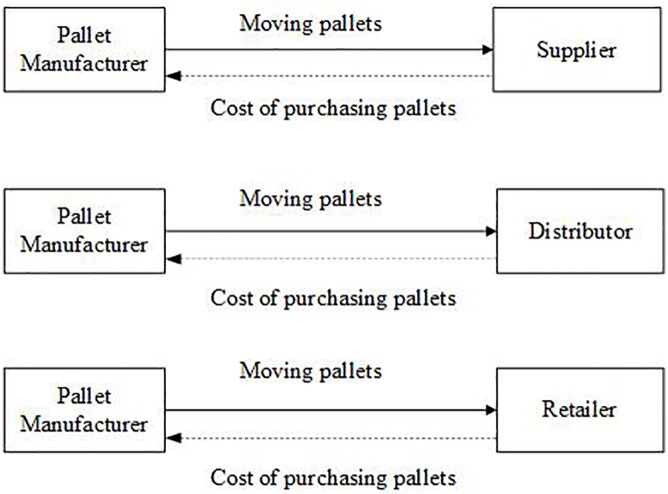
Operations of EMP.

A basic pallet supply chain which consists of a supplier, distributor, and retailer is studied in this paper. As shown in Fig 1, in the EMP system, pallets are not transported with the cargoes. They are only used in a certain company. Suppliers purchase some pallets to load their own cargoes. When having been transported to distributors, cargoes will be unloaded from these pallets. This procedure exists actually in distributors and retailers too.

**Fig 2:**
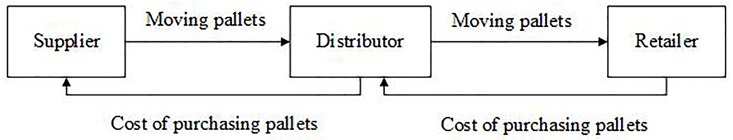
Operations of TPO.

As shown in Fig 2, in the system of TPO, pallets can be moved from suppliers (distributors) to distributors (retailers), but distributors (retailers) have to buy pallets from suppliers (distributors). Retailers should sell these pallets with their cargoes to downstream customers. In our study, we have simplified this process by introducing a three echelons supply chain in which all pallets are sold to virtual recyclers. We assume that the costs of purchasing second-hand pallets from suppliers (distributors) are less than purchasing brand-new ones from pallet manufactures. Distributors (retailers) don’t accept damaged pallets. Damaged pallets have to be delivered to a maintenance station, and they will be put back in to use to the suppliers (distributors) after being repaired.

**Fig 3:**
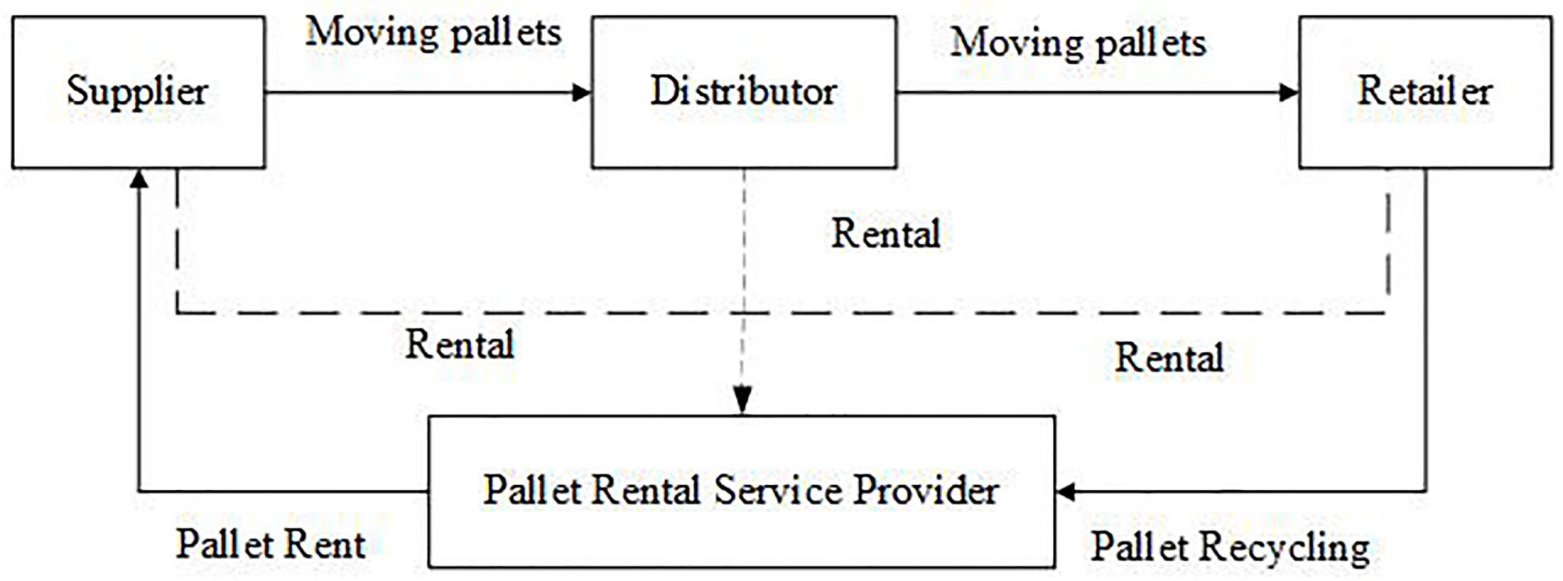
Operations of PR.

As shown in Fig 3, in the system of PR, suppliers rent pallets from a pallet rental service provider. They use these rental pallets to transport cargoes. Cargoes don’t necessarily need to be unloaded from pallets. Distributors can use these pallets on the condition that the rental fee is paid. Retailers can also use these pallets if they pay the rental fee as well. The pallet rental service provider offers services as logistics, recycling, maintenance, and so on. If pallets are lost, supplier (distributor or retailer) has to pay the pallet rental service provider for these lost pallets.

## Cost models

In order to study cost models for the three PMS systems, we have investigated several pallet companies, such as Commonwealth Handling Equipment Pool (the global leader in pallet services), China Merchants Loscam (the largest pallet pooling service provider), Jituo Pool (a pallet-sharing services provider), etc. Some pallets users were also visited, such as Inner Mongolia Yili Industrial Group (the China’s largest dairy company), China Railway Hohhot Group (a subsidiaries company under the jurisdiction of the China Railway), Inner Mongolia Junzheng Energy & Chemical Group (one of the largest energy & chemical company), and so on. These cost models proposed have been validated by the managers of these companies.

### EMP

**Fig 4:**
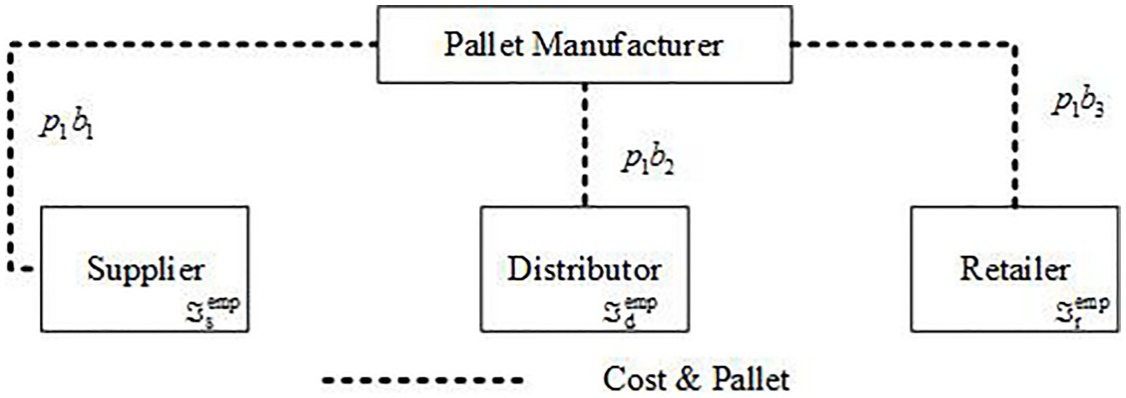
Pallet and cost flow of an EMP system.

Pallets and cost flow of an EMP system is shown in Fig 4.

The total supply chain cost of EMP system (TC^emp^) consists of supplier cost (${\text{C}}_{\text{s}}^{\text{emp}}$), distributor cost (${\text{C}}_{\text{d}}^{\text{emp}}$), and retailer cost (${\text{C}}_{\text{r}}^{\text{emp}}$), as shown in (Eq 1).

$$\text{T}{\text{C}}^{\text{emp}}={\text{C}}_{\text{s}}^{\text{emp}}+{\text{C}}_{\text{d}}^{\text{emp}}+{\text{C}}_{\text{r}}^{\text{emp}}$$ (1)

${\text{C}}_{\text{s}}^{\text{emp}}$, ${\text{C}}_{\text{d}}^{\text{emp}}$, and ${\text{C}}_{\text{r}}^{\text{emp}}$ can be calculated by Eqs (2), (3) and (4), respectively.

$${\text{C}}_{\text{s}}^{\text{emp}}={\text{p}}_{1}{\text{b}}_{1}+{\text{ℑ}}_{\text{s}}^{\text{emp}}$$ (2)

$${\text{C}}_{\text{d}}^{\text{emp}}={\text{p}}_{1}{\text{b}}_{2}+{ℑ}_{\text{d}}^{\text{emp}}$$ (3)

$${\text{C}}_{\text{r}}^{\text{emp}}={\text{p}}_{1}{\text{b}}_{3}+{ℑ}_{\text{r}}^{\text{emp}}$$ (4)

p_1_ is the depreciation value per pallet. b_1_, b_2_, and b_3_are respectively the number of pallets held by a supplier, distributor, and retailer. Pallets are purchased only if a supplier (distributor or retailer) has no pallets available. ${ℑ}_{\text{s}}^{\text{emp}}$, ${ℑ}_{\text{d}}^{\text{emp}}$, and ${ℑ}_{\text{r}}^{\text{emp}}$ are respectively the management and operating cost of supplier, distributor, and retailer which consist of storage cost, loading & unloading cost, and so on.

### TPO

**Fig 5:**
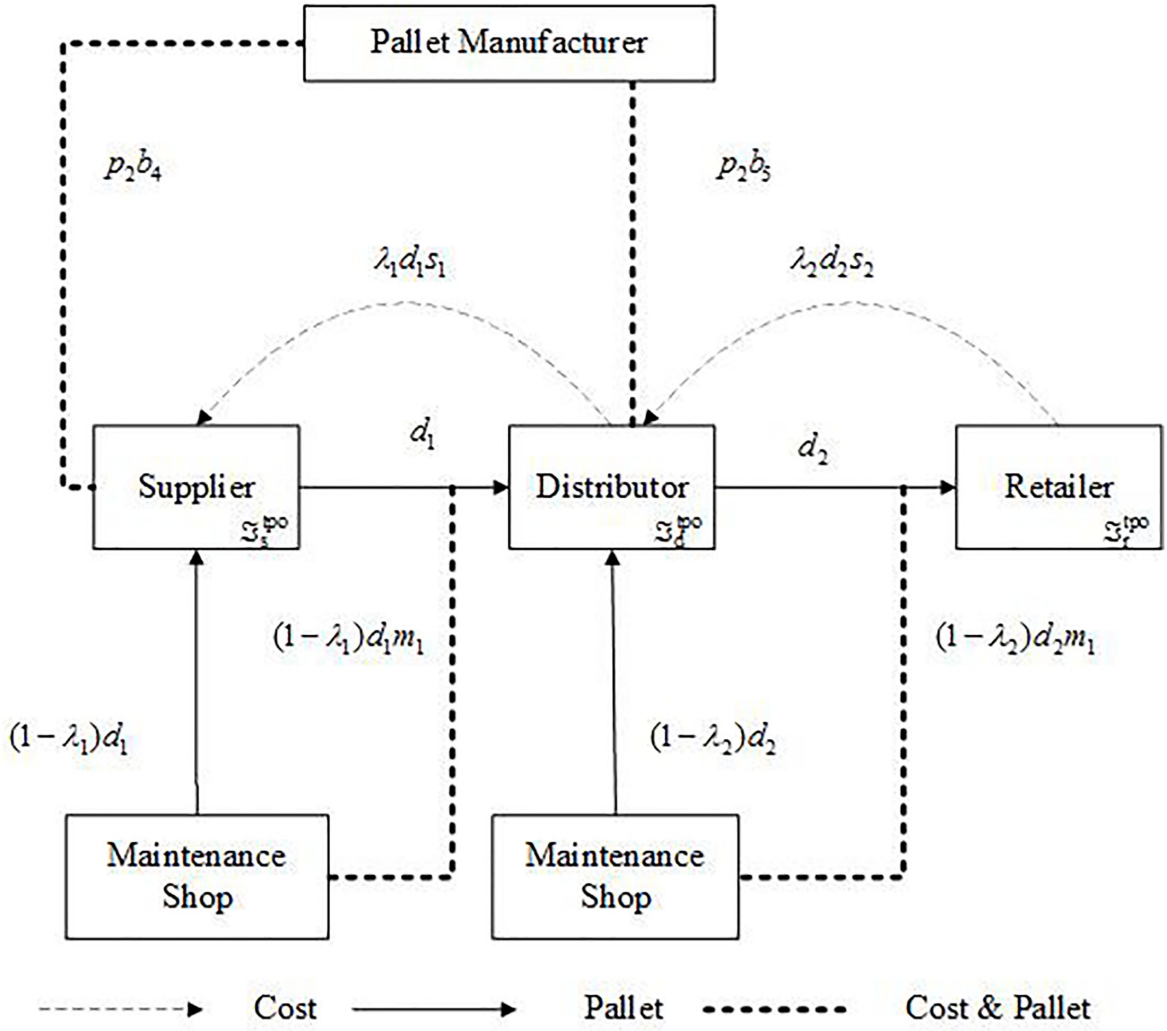
Pallet and cost flow of a TPO system.

Pallets and cost flow of a TPO system is shown in Fig 5.

The total supply chain cost of TPO system (TC^tpo^) consists of supplier cost (${\text{C}}_{\text{s}}^{\text{tpo}}$), distributor cost (${\text{C}}_{\text{d}}^{\text{tpo}}$), and retailer cost (${\text{C}}_{\text{r}}^{\text{tpo}}$), as shown in (Eq 5).

$$\text{T}{\text{C}}^{\text{tpo}}={\text{C}}_{\text{s}}^{\text{tpo}}+{\text{C}}_{\text{d}}^{\text{tpo}}+{\text{C}}_{\text{r}}^{\text{tpo}}$$ (5)

A supplier has to pay for brand-new pallets, pallet maintenance, and management & operating cost. Meanwhile, it can earn reward by selling second-hand pallets to a distributor. ${\text{C}}_{\text{s}}^{\text{tpo}}$ can be calculated by (Eq 6).

$${\text{C}}_{\text{s}}^{\text{tpo}}={\text{p}}_{2}{\text{b}}_{4}-\lambda_{1}{\text{s}}_{1}{\text{d}}_{1}+(1-\lambda_{1}){\text{m}}_{1}{\text{d}}_{1}+{ℑ}_{\text{s}}^{\text{tpo}}$$ (6)

p_2_ is the price of a brand-new pallet. b_4_ (b_4_ = λ_1_d_1_) is the number of brand-new pallets which are purchased by a supplier. Pallets are purchased only if a supplier has not sufficient intact pallets. d_1_is the number of pallets moved from the supplier to a distributor. λ_1_ is the percentage of intact pallets after being moved. The distributor has to pay s_1_ for a second-hand pallet from this supplier. m_1_ is the maintenance cost of a pallet. ${ℑ}_{\text{s}}^{\text{tpo}}$ is a supplier’s management and operating cost.

A distributor has to pay for brand-new pallets, second-hand pallets, pallet maintenance, and management & operating cost. It can also receive income by selling second-hand pallets to a retailer. ${\text{C}}_{\text{d}}^{\text{tpo}}$ can be calculated by (Eq 7).

$${\text{C}}_{\text{d}}^{\text{tpo}}={\text{p}}_{2}{\text{b}}_{5}\text{+}\lambda_{\text{1}}{\text{s}}_{\text{1}}{\text{d}}_{\text{1}}-\lambda_{2}{\text{s}}_{2}{\text{d}}_{2}+(1-\lambda_{2}){\text{m}}_{2}{\text{d}}_{2}+{ℑ}_{\text{d}}^{\text{tpo}}$$ (7)

b_5_ (b_5_ = max{0, λ_2_d_2_−λ_1_d_1_}) is the number of brand-new pallets which are purchased by a distributor. Pallets are purchased only if a distributor has not enough intact pallets. d_2_ is the number of pallets moved from this distributor to a retailer. λ_2_ is the percentage of intact pallets after being moved. The retailer has to pay s_2_ for a second-hand pallet from this distributor. m_2_ is the maintenance cost of a pallet. ${ℑ}_{\text{d}}^{\text{tpo}}$ is a distributor’s management and operating cost.

A retailer has to pay for second-hand pallets and management & operating cost. It can also sell its second-hand pallets to a virtual recycler. ${\text{C}}_{\text{r}}^{\text{tpo}}$ can be calculated by (Eq 8).

$${\text{C}}_{\text{r}}^{\text{tpo}}=\lambda_{2}{\text{d}}_{2}{\text{s}}_{2}\text{-}\lambda_{2}{\text{d}}_{2}{\text{s}}_{3}+{ℑ}_{\text{r}}^{\text{tpo}}$$ (8)

s_3_ is the price of a second-hand pallet sold to a virtual recycler. ${ℑ}_{\text{r}}^{\text{tpo}}$ is a retailer’s management and operating cost.

### PR

**Fig 6:**
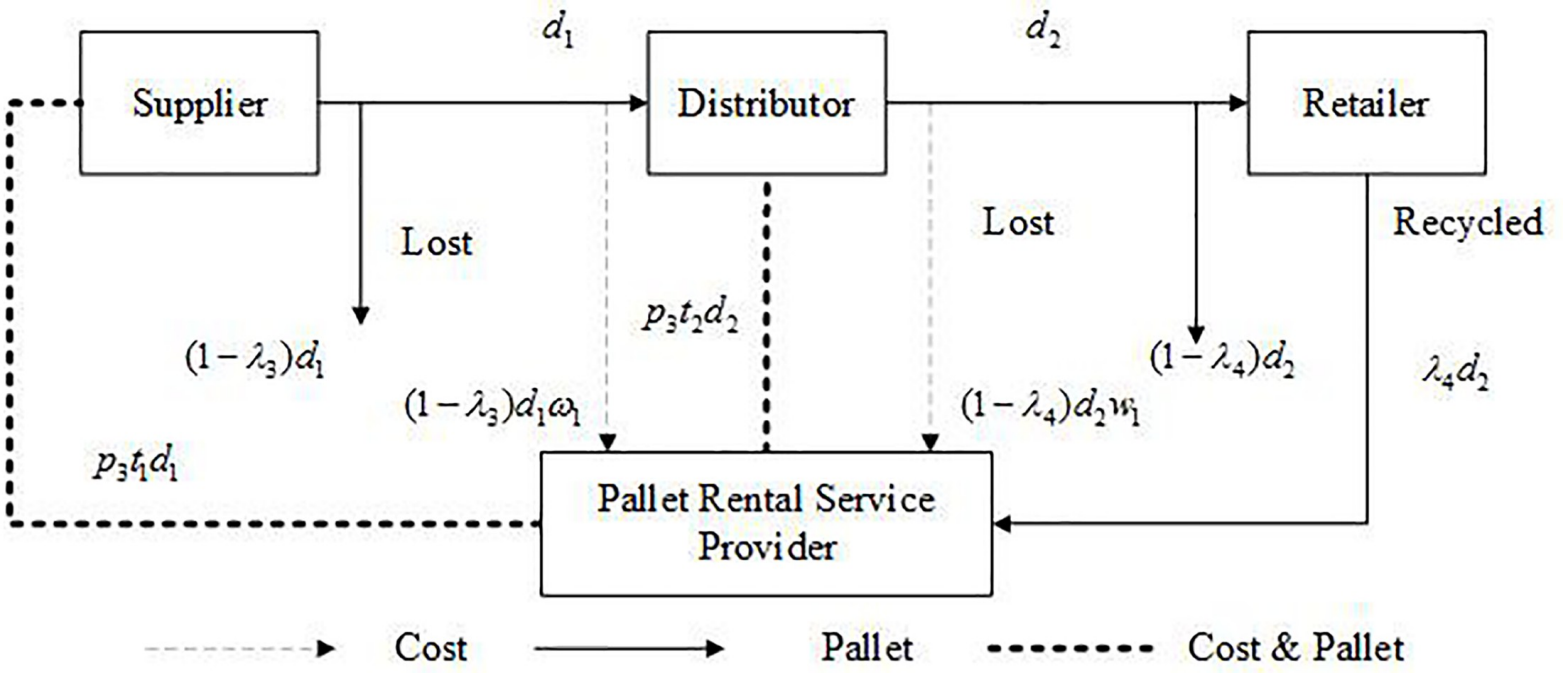
Pallet and cost flow of a PR system.

Pallets and cost flow of a PR system is shown in Fig 6.

The total supply chain cost of PR system (TC^pr^) consists of supplier cost (${\text{C}}_{\text{s}}^{\text{pr}}$) and distributor cost (${\text{C}}_{\text{d}}^{\text{pr}}$), as shown in (Eq 9). In this system, it is assumed the retailer does not need to rent pallets, because pallets will be delivered to pallet rental service provider after cargoes are unloaded.

$$\text{T}{\text{C}}^{\text{p}\text{r}}={\text{C}}_{\text{s}}^{\text{pr}}+{\text{C}}_{\text{d}}^{\text{pr}}$$ (9)

$C_{s}{}^{pr}$ and $C_{d}{}^{pr}$ can be calculated by Eqs (10) and (11), respectively.

$${\text{C}}_{\text{s}}^{\text{pr}}={\text{p}}_{3}^{}{\text{t}}_{1}{\text{d}}_{1}+(1-\lambda_{3}){\text{d}}_{1}{\text{ω}}_{1}$$ (10)

$${\text{C}}_{\text{d}}^{\text{pr}}={\text{p}}_{3}^{}{\text{t}}_{2}{\text{d}}_{2}+(1-\lambda_{4}){\text{d}}_{2}{\text{ω}}_{1}$$ (11)

*p*_3_ is the rental fee of a pallet. *t*_1_(*t*_2_) is the dwell time of pallets in a supplier (distributor). 1-*λ*_3_ (1-*λ*_4_) is the percentage of lost pallets after being moved from a supplier to a distributor (from a distributor to a retailer). *ω*_1_ is the cost per lost pallet (It can be regarded as the cost of purchasing a pallet by pallet rental service providers).

## Simulation and results

AnyLogic is a simulation software which can be used to simulate discrete, continuous, and agent mixed behavior. Since 2003, AnyLogic software has been used in the business simulation of manufacturing, supply chain, logistics, retail, traffic, and aerospace [34]. The operations process of pallet systems with different PMS is simulated by AnyLogic software. Pallet operation processes (purchasing, ownership transfer, maintenance, etc.) are discrete events, so AnyLogic can be used to simulate them.

In order to compare the three PMS, we assume that: (1) A basic pallet supply chain consists of a supplier, distributor, and retailer in any kind of PMS. (2) The inventory control system of all companies is the fixed period ordering. The order for the replenishment of pallets is proposed monthly (30 days). The order quantity varies each time the order is placed. (3) The length of decision period is 100 months. We set this parameter in AnyLogic software as 100 months’ operation for each PMS system. (4) The number of pallets from a supplier to a distributor is equivalent in every pallet system. So is the number of pallets from a distributor to a retailer. (5) In the system of EMP and TPO, entities (suppliers, distributors, or retailers) pay the same price if they purchase the same type of pallet from a manufacture. (6) In the system of PR, customers (suppliers, distributors, or retailers) pay the same rental fee if they rent the same type of pallet from a pallet rental service provider.

A discrete event simulation model is created in Anylogic software. In the Simulation Module, we set time as 100 months. We have called three sub-modules in the Main Module which are Process Modeling Library, Agent, and Analysis. Their functions are illustrated in Tables 1–3.

**Table 1:** Process modeling library.

Process Modeling Library	Function
Source	To indicate pallet manufactures and pallet rental companies. They provide pallets for Service (suppliers, distributors, and retailers).
Service	To indicate suppliers, distributors, and retailers.
Hold	To set the upper bound of pallets moving from suppliers to distributors (or from distributors to retailers).
Delay	To set the lower bound of pallets moving from suppliers to distributors (or from distributors to retailers).
SelectOutput	To set the percentage of damaged pallets or lost pallets.
Sink	To indicate the destination of pallets when they are lost, discarded or being moved to a virtual recycler.

**Table 2:** Agent.

Agent	Function
Event	To control the flow of pallets.
Parameter	To set price, rental fee, lost percentage, and the other parameters.

**Table 3:** Analysis.

Analysis	Function
Plot	To collect and analyze the number of pallets and supply chain cost per month.

### Data collection

The parameters involved in the cost models are valued based on our investigation for several companies as described in Section cost models. We found the management and operating cost of suppliers, distributors, and retailers had no significant difference. Therefore, we set that: ${ℑ}_{\text{s}}^{\text{emp}}={ℑ}_{}^{\text{emp}}\times {\text{b}}_{1}$,${ℑ}_{\text{d}}^{\text{emp}}={ℑ}_{}^{\text{emp}}\times {\text{b}}_{2}$, ${ℑ}_{\text{r}}^{\text{emp}}={ℑ}_{}^{\text{emp}}\times {\text{b}}_{3}$, ${ℑ}_{\text{s}}^{\text{tpo}}={ℑ}_{}^{\text{tpo}}\times {\text{d}}_{1}$,${ℑ}_{\text{d}}^{\text{tpo}}={ℑ}_{}^{\text{tpo}}\times {\text{d}}_{2}$, ${ℑ}_{\text{r}}^{\text{tpo}}={ℑ}_{}^{\text{tpo}}\times {\text{d}}_{2}$. The value of parameters is shown in Table 4.

**Table 4:** Value of parameters.

PMS	Parameter	Value
EMP	${ℑ}_{}^{\text{emp}}$	[0, 15] RMB
	P_1_	[0, 150] RMB
TPO	λ_1_,λ_2_	78%
	${ℑ}_{}^{\text{tpo}}$	[0,15] RMB
	P_2_	[3030, 200] RMB.
	m_1_,m_2_	[0, 30] RMB
	S_3_	[29.1, 194] RMB
	d_1_, d_2_	50
RR	*P*_3_	[0.2, 0.5] RMB
	*λ*_3_,*λ*_4_	80%
	*ω*_1_	[0,200] RMB
	d_1_, d_2_	50
	t_1_, t_2_	30

[a, b] means that the parameter’s value is uniformly distributed between a and b. Because the order for the replenishment of pallets is placed monthly (30 days), the dwell time of pallets in a supplier (distributor) is also 30 days. If we don’t set like that, we found it’s impossible to compare the three PMS. For the same reason, we set d_1_ = d_2_. In the EMP system, the supplier, distributor, and retailer each needs d_1_ pallets to load its own cargoes.

### Results

The total number of pallets from the supplier (distributor) to the distributor (retailer) is shown in Table 5.

**Table 5:** The number of pallets were moved.

PMS	Supplier-Distributor	Distributor -Retailer
EMP	-	-
TPO	5000	5000
PR	5000	5000

In the system of EMP, the supplier, distributor, and retailer each has to purchase 50 brand-new pallets every month because their pallets are disposable.

**Fig 7:**
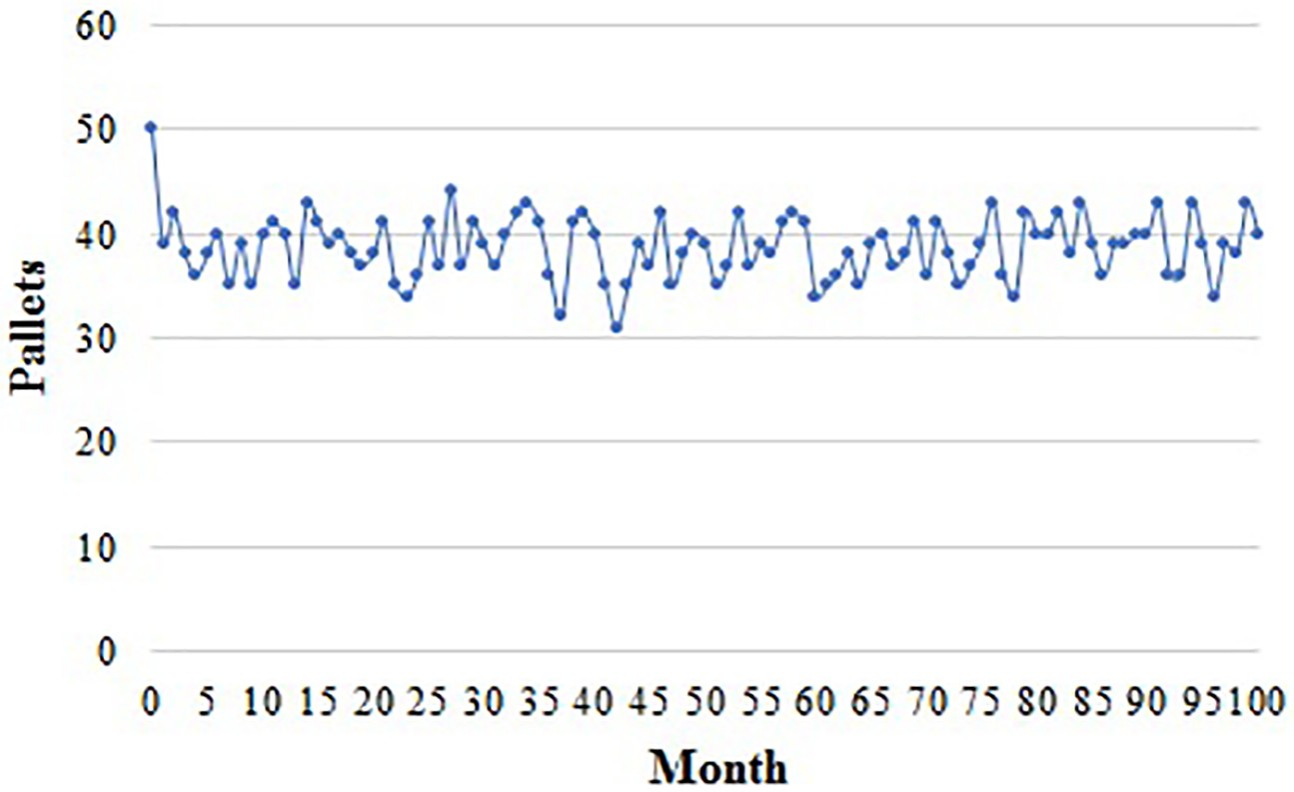
Pallets purchased by the supplier in a TPO system.

**Fig 8:**
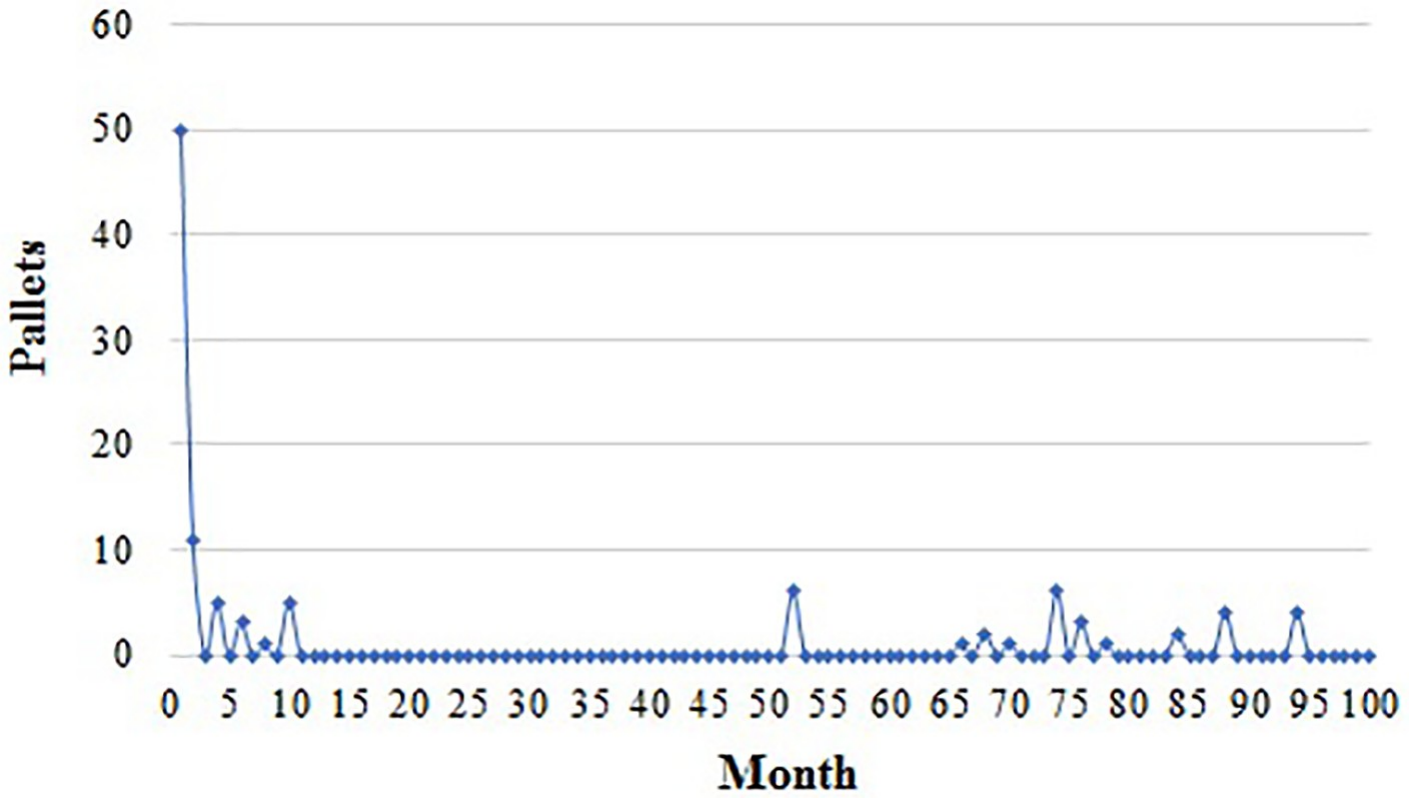
Pallets purchased by the distributor in a TPO system.

In the system of TPO, the number of pallets purchased by the supplier and distributor is shown in Figs 7 and 8. They purchase different quantity every month because they can obtain pallets in approaches like maintenance stations or their partners.

**Fig 9:**
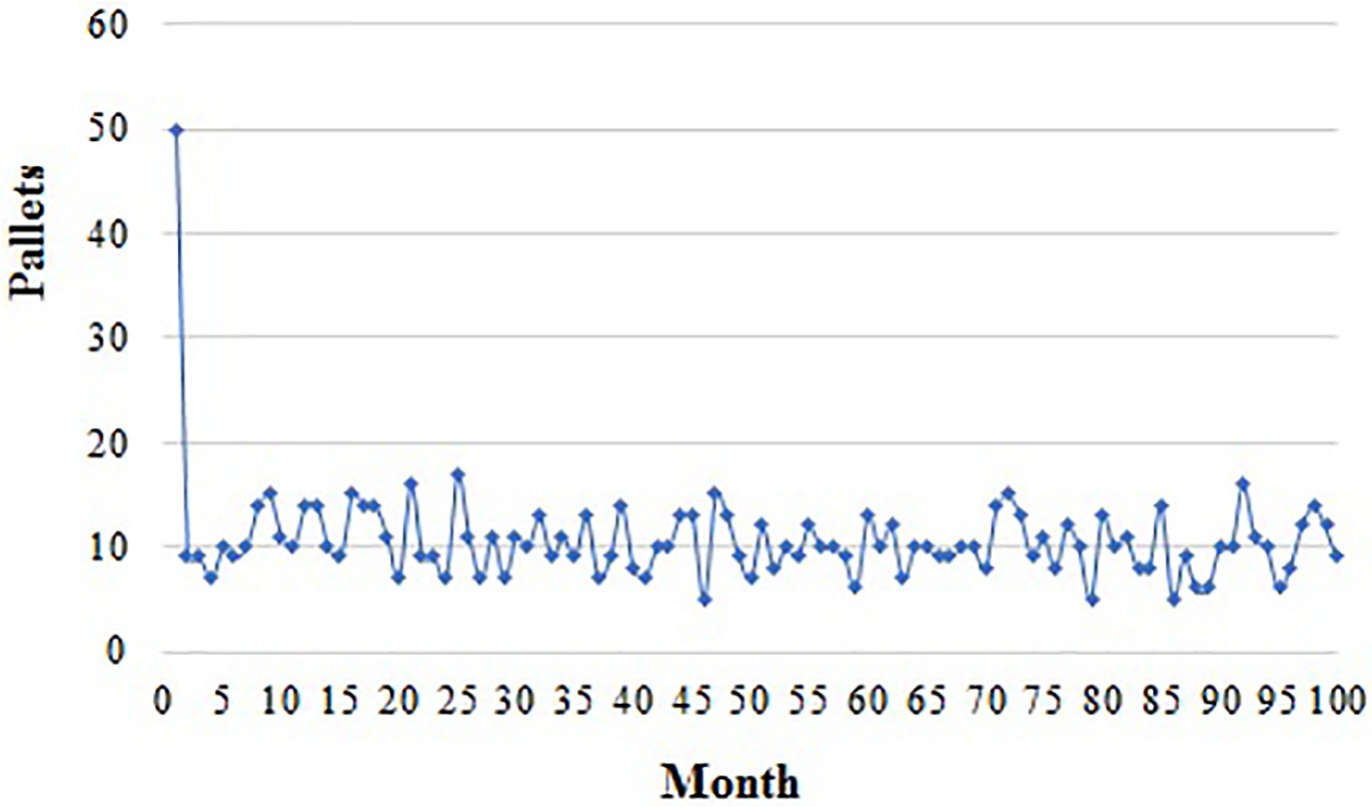
Pallets rented by the distributor.

In the system of PR, the supplier rents 50 pallets from the pallet rental service provider However, as shown in Fig 9, the number of pallets rented by the distributor changes every month because the distributor can receive some pallets from its supplier.

As shown in Table 6, in this case, we found that the total supply chain cost of the TPO system is the lowest, and the PR system is lower than EMP.

**Table 6:** Total supply chain cost.

EMP(RMB)	TPO RMB(RMB)	PR(RMB)
1736744	133968	162093

In order to make sure how operation period affects the cost of each PMS system, we also studied the relationship of operation periods and cost through regression analysis. The models of EMP, TPO, and PR system are respectively shown in Eqs (12), (13) and (14). In these models, y represents the cost, x is the operation period, and R^2^ is coefficient of determination.

$$\text{y}\;=\;17367.44\;\text{x},\;{\text{R}}^{2}=1$$ (12)

$$\text{y}\;=\;1189.7\text{x}\;+\;13717,\;{\text{R}}^{2}=0.9978$$ (13)

$$\text{y}\;=\;1627.3\text{x}\;+\;258.58,\;{\text{R}}^{2}=0.9999$$ (14)

**Fig 10:**
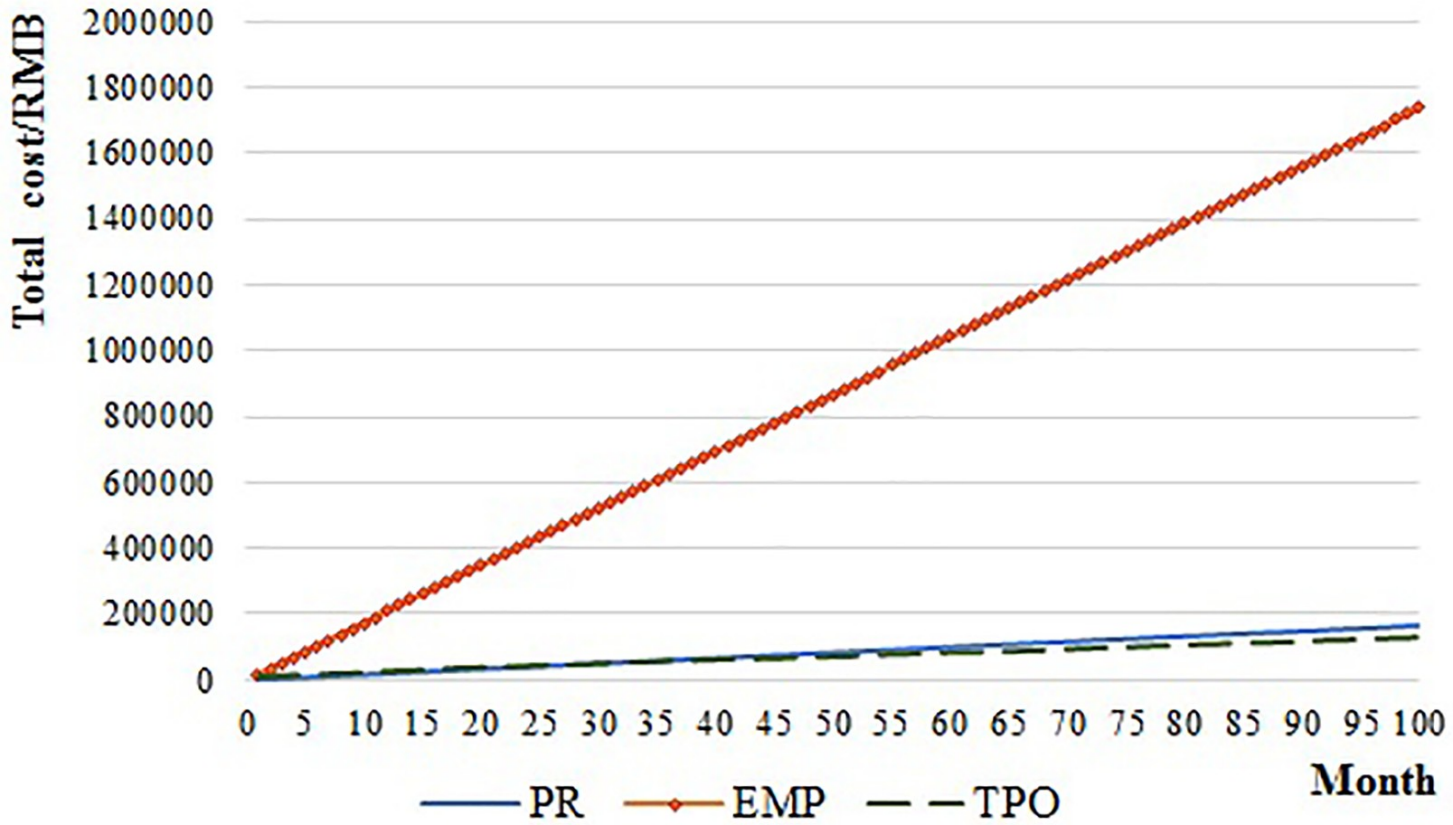
The relationship of operation period and cost.

Fig 10 also shows the relationship of operations period and total cost in this case. EMP cost the most no matter how long the operation period is. When the operation period is shorter than 37 months, the cost of TPO is higher than PR. When the operation period is longer than 37 months, the cost of PR is higher than TPO.

### Sensitivity analysis

The price of pallets (rental fee of pallets), management and operating cost, maintenance cost, and expected cost of lost pallets can seriously affect the simulation results. So, it is necessary for us to analyze the effect of these parameters on selection of PMS by sensitivity analysis. The determination of parameter ranges are based on the field survey as discussed in the Section data collection. We have expanded the parameter ranges in the purpose of obtaining ideal robustness and practicability. For instance, although the rental fee of pallets in PR system proximately ranges from 0.2 RMB to 0.5 RMB in the market, we chose to set this parameter in 0 RMB to 0.5 RMB when implementing our simulation.

#### Price (depreciation value or rental)

**Fig 11:**
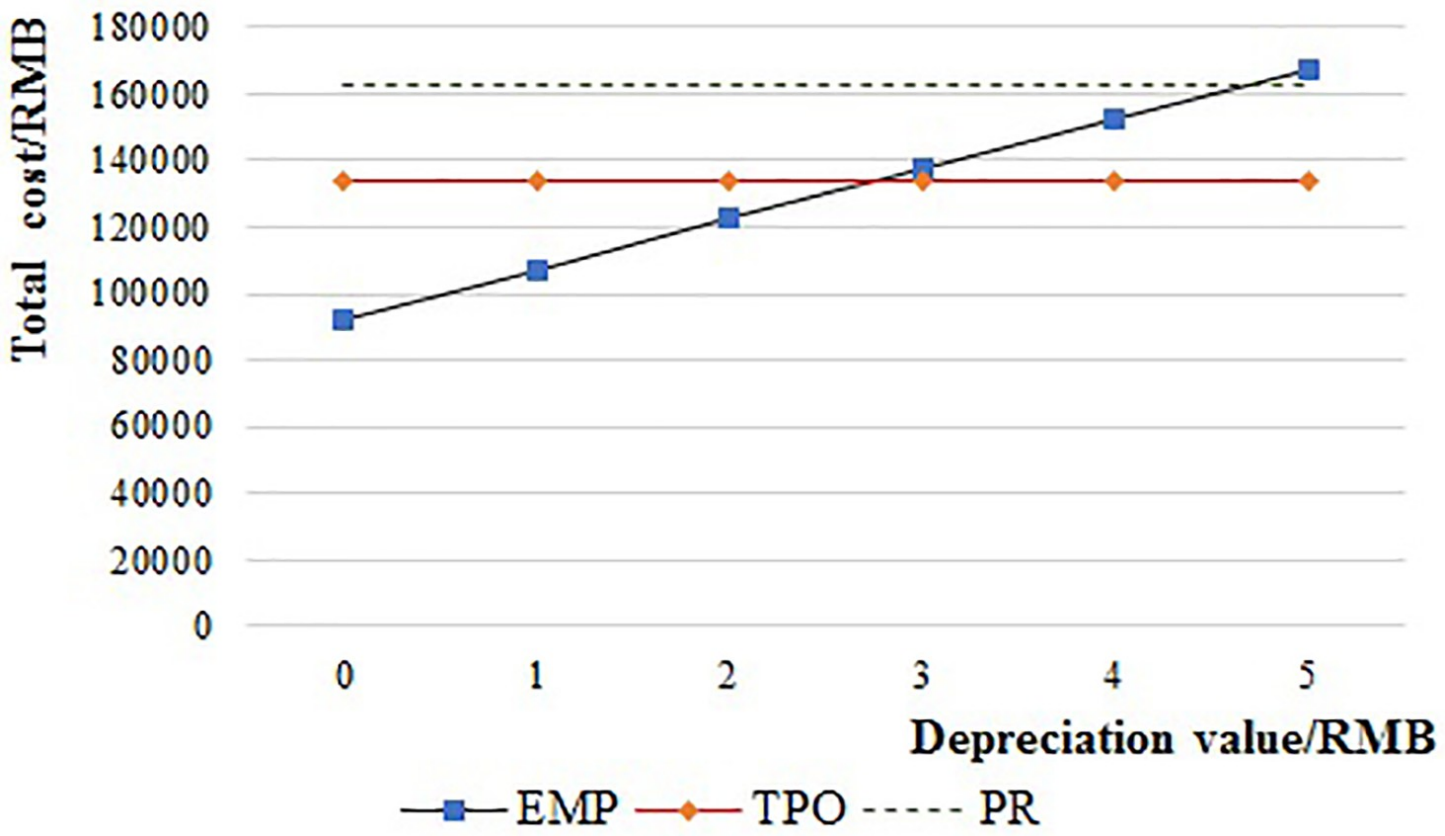
The relationship of the supply chain cost and pallet’s depreciation value of EMP.

Fig 11 shows the total cost of the EMP system when the price of a pallet (depreciation value) is increased from 0 RMB to 150 RMB for each pallet every month. As we found there is no difference on the total cost when the depreciation value is increased from 5 RMB to 150RMB, the relationships in these periods are hence not showed in the Fig 11. The total cost of EMP is the lowest when the depreciation value is less than 2.78 RMB while it becomes the highest when the depreciation value is more than 4.66 RMB. The total cost of EMP is higher than TPO when the depreciation value is between 2.78 RMB and 4.66 RMB whilst lower than PR. In China, according to our survey, the monthly depreciation value for each wooden pallet, plastic pallet, and steel pallet is about 1.3 RMB, 3.3 RMB, and 4.8 RMB, respectively. So, the wooden pallets are the best choice for the EMP system.

**Fig 12:**
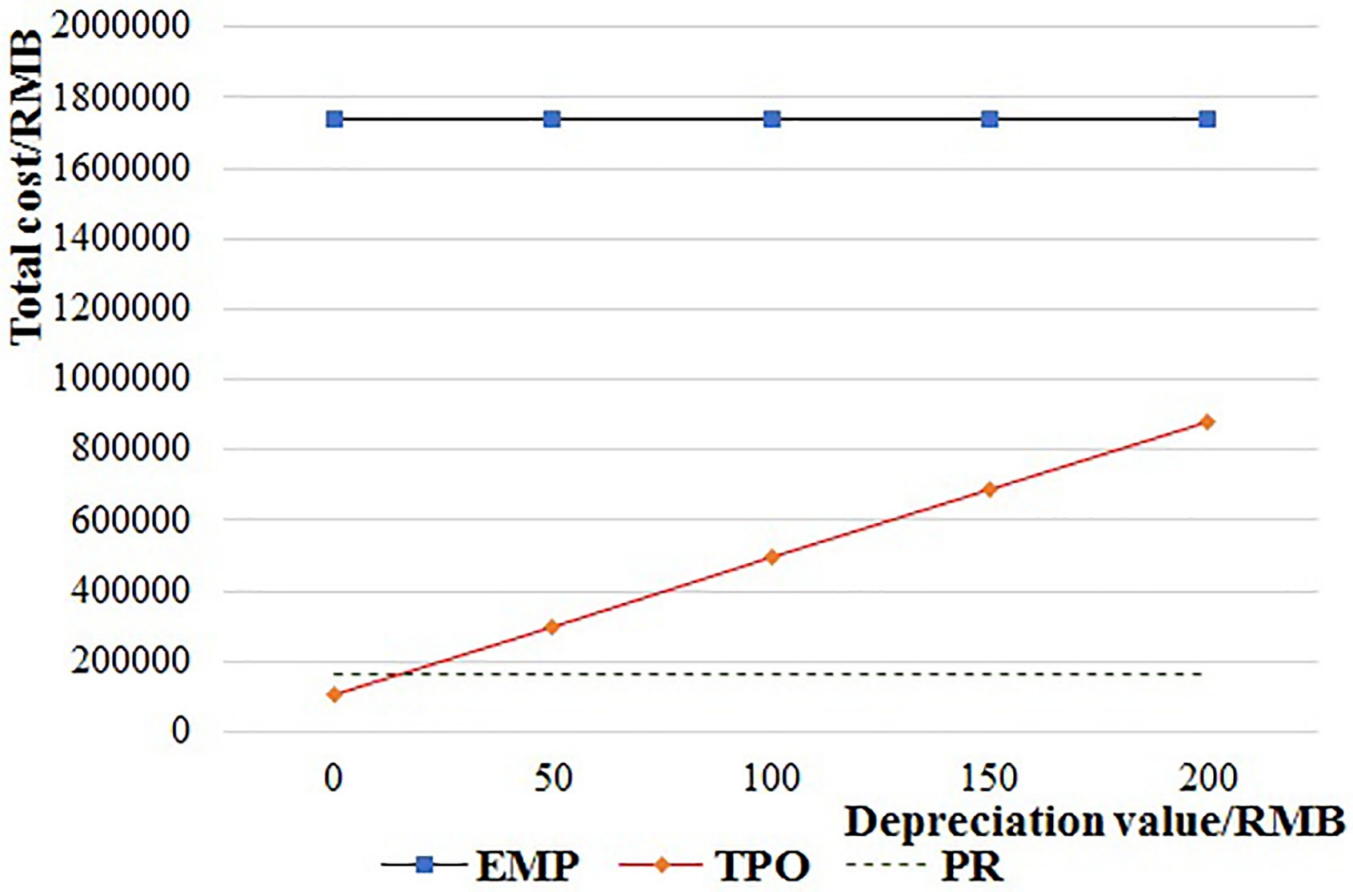
The relationship of the supply chain cost and depreciation value per pallet of TPO.

Both the price of brand-new pallets and second-hand pallets affect the supply chain cost in a TPO system. So, we tested the effect of depreciation value (s = p_2_-s_3_) of pallets. As shown in Fig 12, the total cost of the TPO system is always lower than EMP. The total cost of TPO is lower than PR when the depreciation value per pallet is less than 14.9 RMB, but the total cost of TPO is higher than PR when the depreciation value per pallet is more than 14.9 RMB.

**Fig 13:**
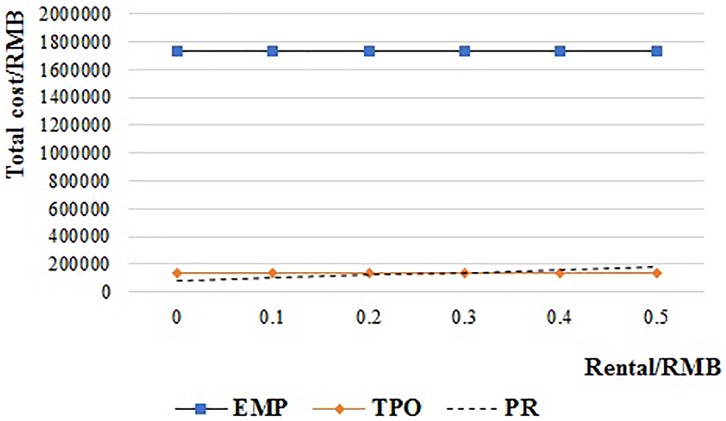
The relationship of the supply chain cost and pallet rental fee of PR.

Fig 13 shows the total cost of the PR system when the rental fee is increased from 0 RMB to 0.5 RMB per pallet. The total cost of PR is always lower than EMP. And it is also lower than TPO when the rental fee is less than 0.265 RMB. The total cost of PR is higher than TPO when the rental fee is more than 0.265 RMB.

#### Management and operating cost

**Fig 14:**
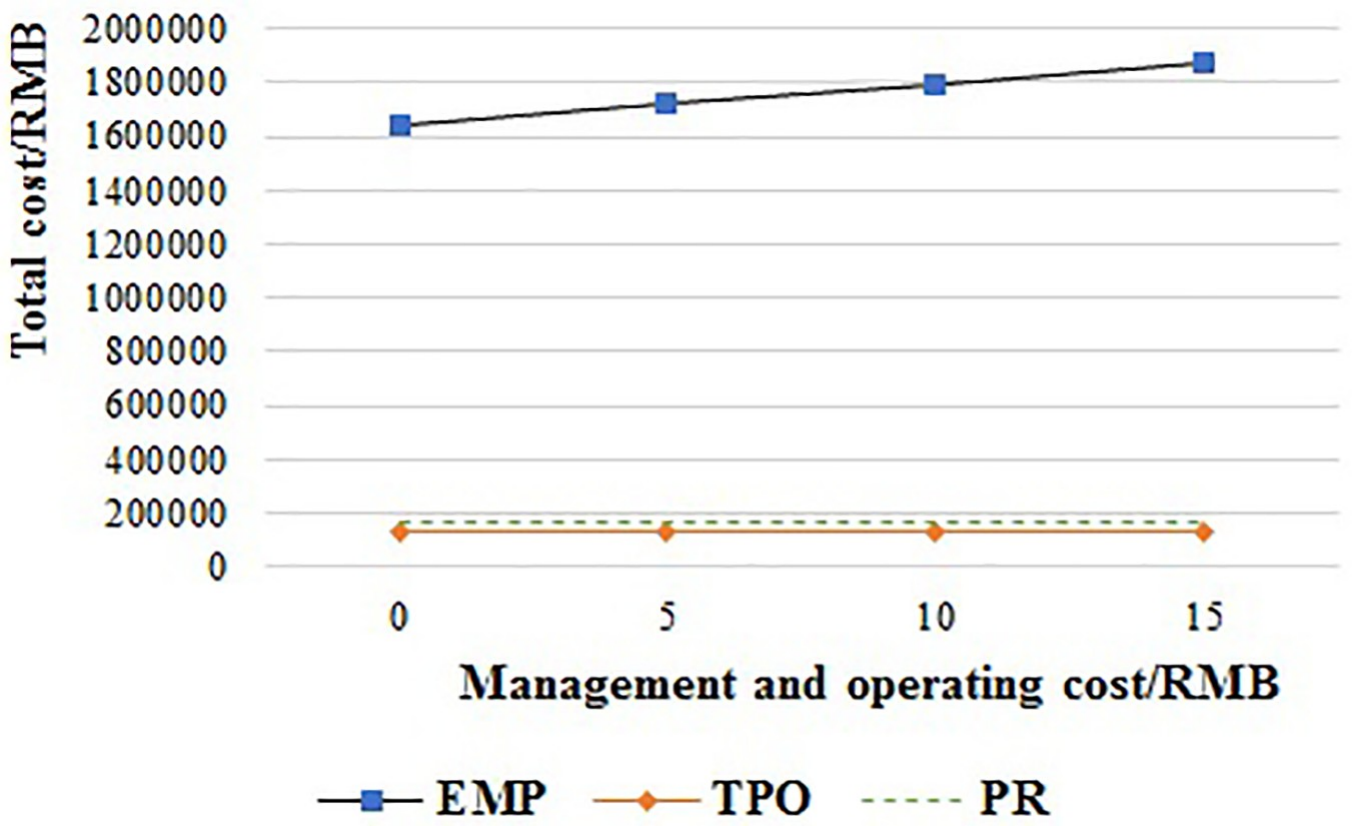
The relationship of the supply chain cost and management and operating cost of EMP.

As shown in Fig 14, the total cost of the EMP system is always the highest of the three PMS, when the management and operating cost are increased from 0 RMB to 15 RMB per pallet.

**Fig 15:**
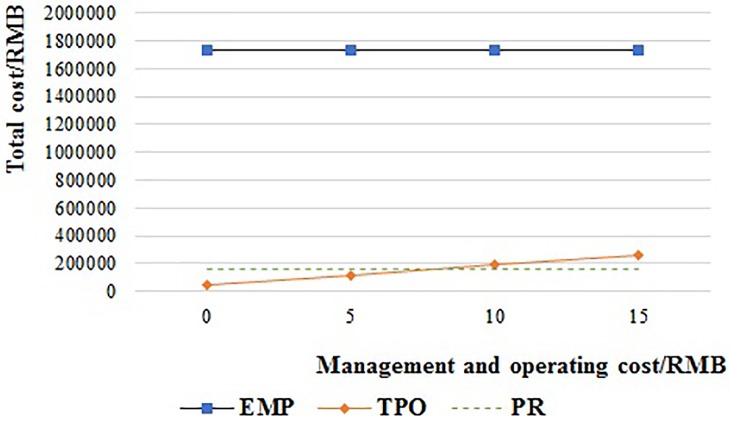
The relationship of the supply chain cost and management and operating cost of TPO.

Fig 15 shows the total cost of the TPO system when the management and operating cost is increased from 0 RMB to 15 RMB per pallet. The total cost of TPO is always lower than EMP. And it is lower than PR when the management and operating cost is less than 8.09 RMB, but higher than PR when the management and operating cost is more than 8.09 RMB.

#### Expected maintenance cost

**Fig 16:**
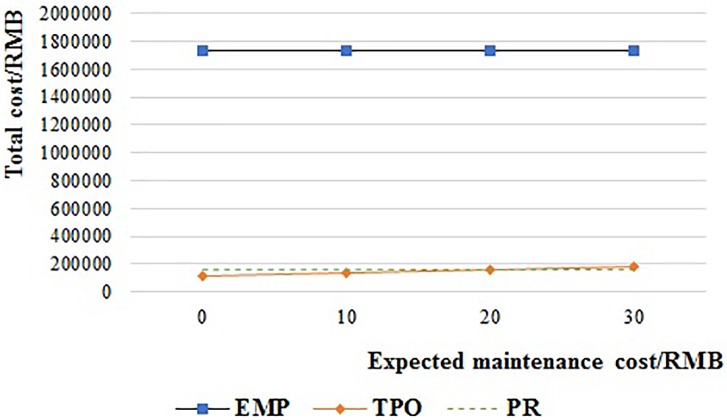
The relationship of the supply chain cost and maintenance cost of TPO.

Fig 16 shows the total cost of the TPO system when the expected maintenance cost ((1-λ_1_)m_1_ and (1-λ_2_)m_2_) is increased from 0 RMB to 30 RMB per pallet. The total cost of TPO is always lower than EMP. And it is lower than PR when the expected maintenance cost is less than 20.2 RMB, but higher than PR when the expected maintenance cost is more than 20.2 RMB.

#### Expected cost of lost

**Fig 17:**
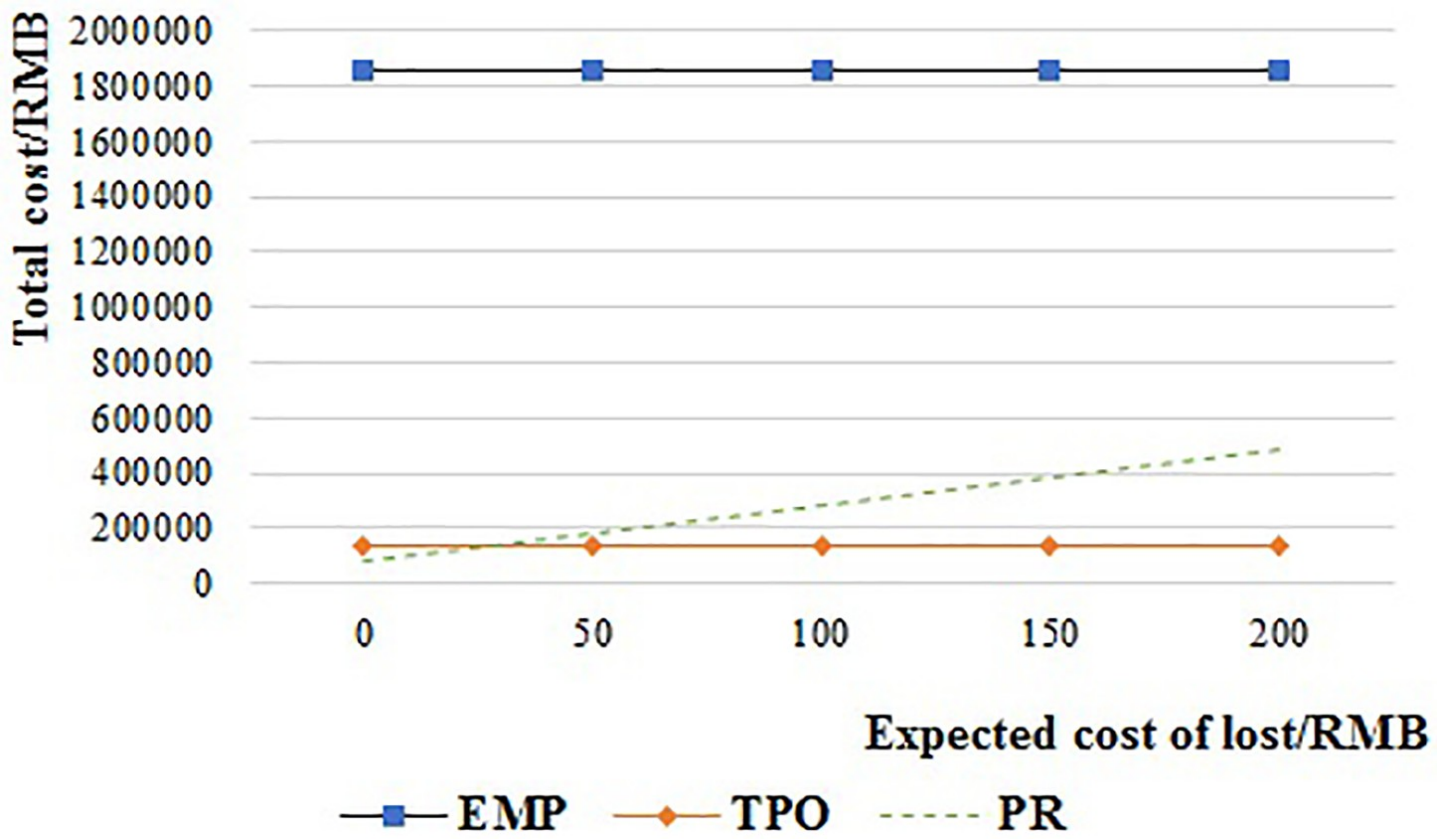
The relationship of the operation cost and lost cost of PR.

Fig 17 shows the operation cost of the PR system when the expected cost per lost pallet ((1-*λ*_3_)*ω*_1_) is increased from 0 RMB to 200 RMB per pallet. The total cost of PR is always lower than EMP. And it is lower than TPO when the expected cost per lost pallet is less than 28 RMB, but higher than TPO when the expected cost per lost pallet is more than 28 RMB.

## Conclusions

The contribution of this work is in developing cost models to understand how to select PMS (EMP, TPO, and PR) from the supply chain perspective based on data collected from filed survey. Although there are some papers on selecting PMS from a single company perspective, we are the first to build models in supply chain perspective as far as we know. Managers should make decisions from the perspective of supply chain instead of a single company, because the competition is no longer between enterprises but between supply chains.

We developed cost models of three kinds of PMS. In order to value parameters involved in the models we proposed, we have investigated several pallet companies and pallets users in China. AnyLogic were used to simulate the operation of pallet management systems. To our deepest and farthest knowledge, AnyLogic is the best software to simulate our models. The effect of parameters on selection of PMS was strictly analyzed by sensitivity analysis, and the validity and utility of our methodologies are convincingly proved.

Based on this research, the selection strategies of PMS can be proposed as follows.

1. Generally speaking, the cost of PR is constantly lower than EMP, and also lower than TPO when the operation period is no more than 37 months. So, PR is the best PMS for most companies if they don’t use pallets for a long time. But they should select TPO in the long run.
2. The price (depreciation value or rental fee) of pallets significantly affects the selection of PMS. The lower price of pallets is, more attractive EMP and TPO become. And the lower rental of pallets is, more attractive the PR is.
3. Managers should try their best to reduce the management and operating cost and maintenance cost if pallets are managed in TPO. Otherwise, they should choose PR.
4. Because it is difficult to reduce the price of pallets, pallet rent service providers should reduce lost rate as much as possible. If their customers cost a lot on lost pallets, they will absolutely choose the other two PMS. Fortunately, more and more pooled pallets are traced with tracking technologies.

Companies should choose PMS based on comparing them with the strategies proposed. Different companies in different situation should choose different PMS.

## Supporting information

S1 Table Data. (DOCX)

S1 Code Simulation of EMP system. (DOCX)

S2 Code Simulation of TPO system. (DOCX)

S3 Code Simulation of PR system. (DOCX)
