# Supplementary material for: Selection of pallet management strategies from the perspective of supply chain cost with Anylogic software
Source: PLoS One. 2019 Jun 6;14(6):e0217995. doi: 10.1371/journal.pone.0217995 (PMC6553775; doi:10.1371/journal.pone.0217995)
Supplement: S1 Table — (DOCX) [file pone.0217995.s001.docx]

**S1 Table. Data**

| Pallet Management Strategy | Parameter | Value | Data Sources |
| --- | --- | --- | --- |
| EMP | $\Im_{\text{s}}^{em\text{p}}$, $\Im_{\text{d}}^{em\text{p}}$, and $\Im_{\text{r}}^{em\text{p}}$is supplier’s, distributor’s, and retailer’s management and operating expenses， respectively | [0, 15] RMB | Inner Mongolia Yili Industrial Group, China Railway Hohhot Group, Inner Mongolia Junzheng Energy & Chemical Group |
|  | $p_{1}$ the depreciation value per pallet | [0, 150] RMB |  |
| TPO | $\lambda_{1}$/$\lambda_{2}$ the percentage of intact pallets after being moved from a supplier to distributor / from a distributor to retailer | 78% | Inner Mongolia Yili Industrial Group, China Railway Hohhot Group, Inner Mongolia Junzheng Energy & Chemical Group |
|  | $\Im_{\text{s}}^{tpo}$, $\Im_{\text{d}}^{\text{tpo}}$, and $\Im_{\text{r}}^{\text{tpo}}$is supplier’s, distributor’s, and retailer’s management and operating expenses ， respectively | [0,15] RMB |  |
|  | $p_{2}$ price of a brand-new pallet | [30, 200] RMB. |  |
|  | $m_{1}$,$m_{2}$ the maintenance cost of a pallet | [0, 30] RMB |  |
|  | $s_{3}$ the price of a second-hand pallet sold to a virtual recycler | [29.1, 194] RMB |  |
|  | $d_{1}$/$d_{2}$ the number of pallets being moved from the supplier to a distributor / from this distributor to a retailer | 50 | assumed |
| RR | $p_{3}$ the rental fee of a pallet | [0.2, 0.5] RMB | China Merchants Loscam, Commonwealth Handling Equipment Pool, Jituo Pool |
|  | $\lambda_{3}$/$\lambda_{4}$ is the percentage of lost pallets after being moved from a supplier to distributor / from a distributor to retailer | 80% |  |
|  | $\omega_{1}$ the cost per lost pallet | [0,200] RMB |  |
|  | $d_{1}$/$d_{2}$ the number of pallets being moved from the supplier to a distributor / from this distributor to a retailer | 50 | assumed |
|  | $t_{1}$/ $t_{2}$ the dwell time of pallets in a supplier / distributor | 30 | assumed |
