# Supplementary material for: Selection of pallet management strategies from the perspective of supply chain cost with Anylogic software
Source: PLoS One. 2019 Jun 6;14(6):e0217995. doi: 10.1371/journal.pone.0217995 (PMC6553775; doi:10.1371/journal.pone.0217995)
Supplement: S3 Code — (DOCX) [file pone.0217995.s004.docx]

**S3 Code. Simulation of PR system**

package 租赁模式;

import java.io.Serializable;

import java.sql.Connection;

import java.sql.SQLException;

import java.util.ArrayDeque;

import java.util.ArrayList;

import java.util.Arrays;

import java.util.Calendar;

import java.util.Collection;

import java.util.Collections;

import java.util.Comparator;

import java.util.Currency;

import java.util.Date;

import java.util.Enumeration;

import java.util.HashMap;

import java.util.HashSet;

import java.util.Hashtable;

import java.util.Iterator;

import java.util.LinkedHashMap;

import java.util.LinkedHashSet;

import java.util.LinkedList;

import java.util.List;

import java.util.ListIterator;

import java.util.Locale;

import java.util.Map;

import java.util.PriorityQueue;

import java.util.Random;

import java.util.Set;

import java.util.SortedMap;

import java.util.SortedSet;

import java.util.Stack;

import java.util.Timer;

import java.util.TreeMap;

import java.util.TreeSet;

import java.util.Vector;

import java.awt.Color;

import java.awt.Font;

import java.awt.Graphics2D;

import java.awt.geom.AffineTransform;

import com.anylogic.engine.connectivity.ResultSet;

import com.anylogic.engine.connectivity.Statement;

import com.anylogic.engine.elements.IElementDescriptor;

import com.anylogic.engine.markup.Network;

import com.anylogic.engine.Position;

import com.anylogic.engine.markup.PedFlowStatistics;

import com.anylogic.engine.markup.DensityMap;

import static java.lang.Math.*;

import static com.anylogic.engine.UtilitiesArray.*;

import static com.anylogic.engine.UtilitiesCollection.*;

import static com.anylogic.engine.presentation.UtilitiesColor.*;

import static com.anylogic.engine.presentation.UtilitiesDrawing.*;

import static com.anylogic.engine.HyperArray.*;

import com.anylogic.engine.*;

import com.anylogic.engine.analysis.*;

import com.anylogic.engine.connectivity.*;

import com.anylogic.engine.database.*;

import com.anylogic.engine.gis.*;

import com.anylogic.engine.markup.*;

import com.anylogic.engine.presentation.*;

import com.anylogic.libraries.processmodeling.*;

import com.mysema.query.Tuple;

import com.mysema.query.sql.SQLBindings;

import static 租赁模式.DBDescriptor.*;

import java.awt.geom.Arc2D;

public class Main extends Agent

{

// 参数

public

double p1;

/**

* 返回参数<code>p1</code>的默认值。

* <i>这个方法不应该被用户调用</i>

*/

@AnyLogicInternalCodegenAPI

public double _p1_DefaultValue_xjal() {

final Main self = this;

return

uniform(0.2,0.5)

;

}

public void set_p1( double p1 ) {

if (p1 == this.p1) {

return;

}

double _oldValue_xjal = this.p1;

this.p1 = p1;

onChange_p1_xjal( _oldValue_xjal );

onChange();

}

/**

* Calls "On change" action for parameter p1.<br>

* Note that 'oldValue' in that action will be unavailable if this method is called by user

* (current parameter value will be passed as 'oldValue').<br>

* Please call <code>set_p1()</code> method instead.

*/

protected void onChange_p1() {

onChange_p1_xjal( p1 );

}

@AnyLogicInternalCodegenAPI

protected void onChange_p1_xjal( double oldValue ) {

}

public

double u;

/**

* 返回参数<code>u</code>的默认值。

* <i>这个方法不应该被用户调用</i>

*/

@AnyLogicInternalCodegenAPI

public double _u_DefaultValue_xjal() {

final Main self = this;

return

0.8

;

}

public void set_u( double u ) {

if (u == this.u) {

return;

}

double _oldValue_xjal = this.u;

this.u = u;

onChange_u_xjal( _oldValue_xjal );

onChange();

}

/**

* Calls "On change" action for parameter u.<br>

* Note that 'oldValue' in that action will be unavailable if this method is called by user

* (current parameter value will be passed as 'oldValue').<br>

* Please call <code>set_u()</code> method instead.

*/

protected void onChange_u() {

onChange_u_xjal( u );

}

@AnyLogicInternalCodegenAPI

protected void onChange_u_xjal( double oldValue ) {

}

public

double x;

/**

* 返回参数<code>x</code>的默认值。

* <i>这个方法不应该被用户调用</i>

*/

@AnyLogicInternalCodegenAPI

public double _x_DefaultValue_xjal() {

final Main self = this;

return

24.101

;

}

public void set_x( double x ) {

if (x == this.x) {

return;

}

double _oldValue_xjal = this.x;

this.x = x;

onChange_x_xjal( _oldValue_xjal );

onChange();

}

/**

* Calls "On change" action for parameter x.<br>

* Note that 'oldValue' in that action will be unavailable if this method is called by user

* (current parameter value will be passed as 'oldValue').<br>

* Please call <code>set_x()</code> method instead.

*/

protected void onChange_x() {

onChange_x_xjal( x );

}

@AnyLogicInternalCodegenAPI

protected void onChange_x_xjal( double oldValue ) {

}

public

double l;

/**

* 返回参数<code>l</code>的默认值。

* <i>这个方法不应该被用户调用</i>

*/

@AnyLogicInternalCodegenAPI

public double _l_DefaultValue_xjal() {

final Main self = this;

return

uniform(0,200)

;

}

public void set_l( double l ) {

if (l == this.l) {

return;

}

double _oldValue_xjal = this.l;

this.l = l;

onChange_l_xjal( _oldValue_xjal );

onChange();

}

/**

* Calls "On change" action for parameter l.<br>

* Note that 'oldValue' in that action will be unavailable if this method is called by user

* (current parameter value will be passed as 'oldValue').<br>

* Please call <code>set_l()</code> method instead.

*/

protected void onChange_l() {

onChange_l_xjal( l );

}

@AnyLogicInternalCodegenAPI

protected void onChange_l_xjal( double oldValue ) {

}

@Override

public void setParametersToDefaultValues() {

super.setParametersToDefaultValues();

p1 = _p1_DefaultValue_xjal();

u = _u_DefaultValue_xjal();

x = _x_DefaultValue_xjal();

l = _l_DefaultValue_xjal();

}

@Override

public boolean setParameter(String _name_xjal, Object _value_xjal, boolean _callOnChange_xjal) {

switch ( _name_xjal ) {

case "p1":

if ( _callOnChange_xjal ) {

set_p1( ((Number) _value_xjal).doubleValue() );

} else {

p1 = ((Number) _value_xjal).doubleValue();

}

return true;

case "u":

if ( _callOnChange_xjal ) {

set_u( ((Number) _value_xjal).doubleValue() );

} else {

u = ((Number) _value_xjal).doubleValue();

}

return true;

case "x":

if ( _callOnChange_xjal ) {

set_x( ((Number) _value_xjal).doubleValue() );

} else {

x = ((Number) _value_xjal).doubleValue();

}

return true;

case "l":

if ( _callOnChange_xjal ) {

set_l( ((Number) _value_xjal).doubleValue() );

} else {

l = ((Number) _value_xjal).doubleValue();

}

return true;

default:

return super.setParameter( _name_xjal, _value_xjal, _callOnChange_xjal );

}

}

@Override

public <T> T getParameter(String _name_xjal) {

Object _result_xjal;

switch ( _name_xjal ) {

case "p1": _result_xjal = p1; break;

case "u": _result_xjal = u; break;

case "x": _result_xjal = x; break;

case "l": _result_xjal = l; break;

default: _result_xjal = super.getParameter( _name_xjal ); break;

}

return (T) _result_xjal;

}

@AnyLogicInternalCodegenAPI

private static String[] _parameterNames_xjal;

@Override

public String[] getParameterNames() {

String[] result = _parameterNames_xjal;

if (result == null) {

List<String> list = new ArrayList<>( Arrays.asList( super.getParameterNames() ) );

list.add( "p1" );

list.add( "u" );

list.add( "x" );

list.add( "l" );

result = list.toArray( new String[ list.size() ] );

_parameterNames_xjal = result;

}

return result;

}

@AnyLogicInternalCodegenAPI

private static Map<String, IElementDescriptor> elementDesciptors_xjal = null;

@AnyLogicInternalCodegenAPI

@Override

public Map<String, IElementDescriptor> getElementDesciptors() {

if (elementDesciptors_xjal == null) {

elementDesciptors_xjal = createElementDescriptors(super.getElementDesciptors(), Main.class);

}

return elementDesciptors_xjal;

}

@AnyLogicCustomProposalPriority(type = AnyLogicCustomProposalPriority.Type.STATIC_ELEMENT)

public static final Scale scale = new Scale( 10.0 );

@Override

public Scale getScale() {

return scale;

}

// 事件

public EventTimeout event = new EventTimeout(this);

public EventTimeout event1 = new EventTimeout(this);

public EventTimeout event2 = new EventTimeout(this);

public EventTimeout event3 = new EventTimeout(this);

@AnyLogicInternalCodegenAPI

public EventTimeout _plot_autoUpdateEvent_xjal = new EventTimeout(this);

@AnyLogicInternalCodegenAPI

public EventTimeout _plot1_autoUpdateEvent_xjal = new EventTimeout(this);

@AnyLogicInternalCodegenAPI

public EventTimeout _plot3_autoUpdateEvent_xjal = new EventTimeout(this);

@AnyLogicInternalCodegenAPI

public EventTimeout _plot2_autoUpdateEvent_xjal = new EventTimeout(this);

@AnyLogicInternalCodegenAPI

public EventTimeout _plot4_autoUpdateEvent_xjal = new EventTimeout(this);

@Override

@AnyLogicInternalCodegenAPI

public String getNameOf( EventTimeout _e ) {

if( _e == event ) return "event";

if( _e == event1 ) return "event1";

if( _e == event2 ) return "event2";

if( _e == event3 ) return "event3";

if( _e == _plot_autoUpdateEvent_xjal ) return "plot auto update event";

if( _e == _plot1_autoUpdateEvent_xjal ) return "plot1 auto update event";

if( _e == _plot3_autoUpdateEvent_xjal ) return "plot3 auto update event";

if( _e == _plot2_autoUpdateEvent_xjal ) return "plot2 auto update event";

if( _e == _plot4_autoUpdateEvent_xjal ) return "plot4 auto update event";

return super.getNameOf( _e );

}

@Override

@AnyLogicInternalCodegenAPI

public EventTimeout.Mode getModeOf( EventTimeout _e ) {

if ( _e == event ) return EVENT_TIMEOUT_MODE_CYCLIC;

if ( _e == event1 ) return EVENT_TIMEOUT_MODE_CYCLIC;

if ( _e == event2 ) return EVENT_TIMEOUT_MODE_CYCLIC;

if ( _e == event3 ) return EVENT_TIMEOUT_MODE_CYCLIC;

if ( _e == _plot_autoUpdateEvent_xjal ) return EVENT_TIMEOUT_MODE_CYCLIC;

if ( _e == _plot1_autoUpdateEvent_xjal ) return EVENT_TIMEOUT_MODE_CYCLIC;

if ( _e == _plot3_autoUpdateEvent_xjal ) return EVENT_TIMEOUT_MODE_CYCLIC;

if ( _e == _plot2_autoUpdateEvent_xjal ) return EVENT_TIMEOUT_MODE_CYCLIC;

if ( _e == _plot4_autoUpdateEvent_xjal ) return EVENT_TIMEOUT_MODE_CYCLIC;

return super.getModeOf( _e );

}

@Override

@AnyLogicInternalCodegenAPI

public double getFirstOccurrenceTime( EventTimeout _e ) {

double _t;

if ( _e == event ) {

_t =

1

;

_t = toModelTime( _t, MONTH );

return _t;

}

if ( _e == event1 ) {

_t =

1

;

_t = toModelTime( _t, MONTH );

return _t;

}

if ( _e == event2 ) {

_t =

0

;

_t = toModelTime( _t, MONTH );

return _t;

}

if ( _e == event3 ) {

_t =

0

;

_t = toModelTime( _t, MONTH );

return _t;

}

if ( _e == _plot_autoUpdateEvent_xjal ) {

_t =

0

;

_t = toModelTime( _t, MONTH );

return _t;

}

if ( _e == _plot1_autoUpdateEvent_xjal ) {

_t =

0

;

_t = toModelTime( _t, MONTH );

return _t;

}

if ( _e == _plot3_autoUpdateEvent_xjal ) {

_t =

1

;

_t = toModelTime( _t, DAY );

return _t;

}

if ( _e == _plot2_autoUpdateEvent_xjal ) {

_t =

1

;

_t = toModelTime( _t, DAY );

return _t;

}

if ( _e == _plot4_autoUpdateEvent_xjal ) {

_t =

0

;

_t = toModelTime( _t, MONTH );

return _t;

}

return super.getFirstOccurrenceTime( _e );

}

@Override

@AnyLogicInternalCodegenAPI

public double evaluateTimeoutOf( EventTimeout _e ) {

double _t;

if( _e == event) {

_t =

1

;

_t = toModelTime( _t, MONTH );

return _t;

}

if( _e == event1) {

_t =

1

;

_t = toModelTime( _t, MONTH );

return _t;

}

if( _e == event2) {

_t =

1

;

_t = toModelTime( _t, MONTH );

return _t;

}

if( _e == event3) {

_t =

1

;

_t = toModelTime( _t, SECOND );

return _t;

}

if( _e == _plot_autoUpdateEvent_xjal) {

_t =

1

;

_t = toModelTime( _t, DAY );

return _t;

}

if( _e == _plot1_autoUpdateEvent_xjal) {

_t =

1

;

_t = toModelTime( _t, DAY );

return _t;

}

if( _e == _plot3_autoUpdateEvent_xjal) {

_t =

1

;

_t = toModelTime( _t, MONTH );

return _t;

}

if( _e == _plot2_autoUpdateEvent_xjal) {

_t =

1

;

_t = toModelTime( _t, MONTH );

return _t;

}

if( _e == _plot4_autoUpdateEvent_xjal) {

_t =

1

;

_t = toModelTime( _t, MONTH );

return _t;

}

return super.evaluateTimeoutOf( _e );

}

@Override

@AnyLogicInternalCodegenAPI

public void executeActionOf( EventTimeout _e ) {

if ( _e == event ) {

EventTimeout self = _e;

Manufacturer0.inject(50);

;

return;

}

if ( _e == event1 ) {

EventTimeout self = _e;

Manufacturer1.inject(delay2.size());

;

return;

}

if ( _e == event2 ) {

EventTimeout self = _e;

hold.unblock();

hold1.unblock();

;

return;

}

if ( _e == event3 ) {

EventTimeout self = _e;

if(delay1.size()==50)

{hold1.block();delay1.stopDelayForAll();}

if(delay.size()==50)

{hold.block();delay.stopDelayForAll();}

;

return;

}

if ( _e == _plot_autoUpdateEvent_xjal ) {

plot.updateData();

return;

}

if ( _e == _plot1_autoUpdateEvent_xjal ) {

plot1.updateData();

return;

}

if ( _e == _plot3_autoUpdateEvent_xjal ) {

plot3.updateData();

return;

}

if ( _e == _plot2_autoUpdateEvent_xjal ) {

plot2.updateData();

return;

}

if ( _e == _plot4_autoUpdateEvent_xjal ) {

plot4.updateData();

return;

}

super.executeActionOf( _e );

}

/** Internal constant, shouldn't be accessed by user */

@AnyLogicInternalCodegenAPI

protected static final short _STATECHART_ELEMENT_NEXT_ID_xjal = 0;

// 嵌入对象

public com.anylogic.libraries.processmodeling.Service<

Agent

> Supplier0;

public com.anylogic.libraries.processmodeling.Service<

Agent

> Distributor0;

public com.anylogic.libraries.processmodeling.Service<

Agent

> Retailer0;

public com.anylogic.libraries.processmodeling.SelectOutput<

Agent

> selectOutput;

public com.anylogic.libraries.processmodeling.Sink<

Agent

> sink;

public com.anylogic.libraries.processmodeling.SelectOutput<

Agent

> selectOutput1;

public com.anylogic.libraries.processmodeling.Sink<

Agent

> sink1;

public com.anylogic.libraries.processmodeling.Sink<

Agent

> sink2;

public com.anylogic.libraries.processmodeling.Source<

Agent

> Manufacturer0;

public com.anylogic.libraries.processmodeling.Source<

Agent

> Manufacturer1;

public com.anylogic.libraries.processmodeling.Hold<

Agent

> hold;

public com.anylogic.libraries.processmodeling.Hold<

Agent

> hold1;

public com.anylogic.libraries.processmodeling.Delay<

Agent

> delay1;

public com.anylogic.libraries.processmodeling.Delay<

Agent

> delay;

public com.anylogic.libraries.processmodeling.Delay<

Agent

> delay2;

public String getNameOf( Agent ao ) {

if ( ao == Supplier0 ) return "Supplier0";

if ( ao == Distributor0 ) return "Distributor0";

if ( ao == Retailer0 ) return "Retailer0";

if ( ao == selectOutput ) return "selectOutput";

if ( ao == sink ) return "sink";

if ( ao == selectOutput1 ) return "selectOutput1";

if ( ao == sink1 ) return "sink1";

if ( ao == sink2 ) return "sink2";

if ( ao == Manufacturer0 ) return "Manufacturer0";

if ( ao == Manufacturer1 ) return "Manufacturer1";

if ( ao == hold ) return "hold";

if ( ao == hold1 ) return "hold1";

if ( ao == delay1 ) return "delay1";

if ( ao == delay ) return "delay";

if ( ao == delay2 ) return "delay2";

return super.getNameOf( ao );

}

public AgentAnimationSettings getAnimationSettingsOf( Agent ao ) {

return super.getAnimationSettingsOf( ao );

}

public String getNameOf( AgentList<?> aolist ) {

return super.getNameOf( aolist );

}

public AgentAnimationSettings getAnimationSettingsOf( AgentList<?> aolist ) {

return super.getAnimationSettingsOf( aolist );

}

/**

* 创建嵌入对象实例<br>

* <i>这个方法不应该被用户调用</i>

*/

protected com.anylogic.libraries.processmodeling.Service<Agent> instantiate_Supplier0_xjal() {

com.anylogic.libraries.processmodeling.Service<Agent> _result_xjal = new com.anylogic.libraries.processmodeling.Service<Agent>( getEngine(), this, null ) {

@Override

public com.anylogic.libraries.processmodeling.ResourcePool[][] resourceSets( Agent agent ) {

return _Supplier0_resourceSets_xjal( this, agent );

}

@Override

public double delayTime( Agent agent ) {

return _Supplier0_delayTime_xjal( this, agent );

}

@AnyLogicInternalCodegenAPI

public TimeUnits getUnitsForCodeOf_delayTime() {

return DAY;

}

/**

* 这个数字在这里是出于模型快照存储的目的。它不应该被用户修改。

*/

@AnyLogicInternalCodegenAPI

private static final long serialVersionUID = -6773233648279431412L;

};

return _result_xjal;

}

/**

* 设置嵌入对象实例的参数<br>

* 这个方法不应该被用户调用

*/

private void setupParameters_Supplier0_xjal( final com.anylogic.libraries.processmodeling.Service<Agent> self ) {

setupParameters_Supplier0_xjal( self, null );

}

/**

* 设置嵌入对象实例<br>

* 这个方法不应该被用户调用

*/

@AnyLogicInternalCodegenAPI

private void create_Supplier0_xjal( com.anylogic.libraries.processmodeling.Service<Agent> self ) {

create_Supplier0_xjal(self, null );

}

/**

* 设置嵌入对象实例的参数<br>

* 这个方法不应该被用户调用

*/

private void setupParameters_Supplier0_xjal( final com.anylogic.libraries.processmodeling.Service<Agent> self, TableInput _t ) {

self.seizeFromOnePool = self._seizeFromOnePool_DefaultValue_xjal();

self.queueCapacity = self._queueCapacity_DefaultValue_xjal();

self.maximumCapacity =

true

;

self.destinationType = self._destinationType_DefaultValue_xjal();

self.entityLocationQueue = self._entityLocationQueue_DefaultValue_xjal();

self.entityLocationDelay = self._entityLocationDelay_DefaultValue_xjal();

self.suspendResumeEntities = self._suspendResumeEntities_DefaultValue_xjal();

self.customizeResourceChoice = self._customizeResourceChoice_DefaultValue_xjal();

self.enableTimeout = self._enableTimeout_DefaultValue_xjal();

self.enablePreemption = self._enablePreemption_DefaultValue_xjal();

self.restoreEntityLocationOnExit = self._restoreEntityLocationOnExit_DefaultValue_xjal();

self.forceStatisticsCollection =

true

;

}

/**

* 设置嵌入对象实例<br>

* 这个方法不应该被用户调用

*/

@AnyLogicInternalCodegenAPI

private void create_Supplier0_xjal( com.anylogic.libraries.processmodeling.Service<Agent> self, TableInput _t ) {

self.create();

}

/**

* 创建嵌入对象实例<br>

* <i>这个方法不应该被用户调用</i>

*/

protected com.anylogic.libraries.processmodeling.Service<Agent> instantiate_Distributor0_xjal() {

com.anylogic.libraries.processmodeling.Service<Agent> _result_xjal = new com.anylogic.libraries.processmodeling.Service<Agent>( getEngine(), this, null ) {

@Override

public double delayTime( Agent agent ) {

return _Distributor0_delayTime_xjal( this, agent );

}

@AnyLogicInternalCodegenAPI

public TimeUnits getUnitsForCodeOf_delayTime() {

return DAY;

}

/**

* 这个数字在这里是出于模型快照存储的目的。它不应该被用户修改。

*/

@AnyLogicInternalCodegenAPI

private static final long serialVersionUID = -6773233648279453155L;

};

return _result_xjal;

}

/**

* 设置嵌入对象实例的参数<br>

* 这个方法不应该被用户调用

*/

private void setupParameters_Distributor0_xjal( final com.anylogic.libraries.processmodeling.Service<Agent> self ) {

setupParameters_Distributor0_xjal( self, null );

}

/**

* 设置嵌入对象实例<br>

* 这个方法不应该被用户调用

*/

@AnyLogicInternalCodegenAPI

private void create_Distributor0_xjal( com.anylogic.libraries.processmodeling.Service<Agent> self ) {

create_Distributor0_xjal(self, null );

}

/**

* 设置嵌入对象实例的参数<br>

* 这个方法不应该被用户调用

*/

private void setupParameters_Distributor0_xjal( final com.anylogic.libraries.processmodeling.Service<Agent> self, TableInput _t ) {

self.seizeFromOnePool = self._seizeFromOnePool_DefaultValue_xjal();

self.queueCapacity = self._queueCapacity_DefaultValue_xjal();

self.maximumCapacity =

true

;

self.destinationType = self._destinationType_DefaultValue_xjal();

self.entityLocationQueue = self._entityLocationQueue_DefaultValue_xjal();

self.entityLocationDelay = self._entityLocationDelay_DefaultValue_xjal();

self.suspendResumeEntities = self._suspendResumeEntities_DefaultValue_xjal();

self.customizeResourceChoice = self._customizeResourceChoice_DefaultValue_xjal();

self.enableTimeout = self._enableTimeout_DefaultValue_xjal();

self.enablePreemption = self._enablePreemption_DefaultValue_xjal();

self.restoreEntityLocationOnExit = self._restoreEntityLocationOnExit_DefaultValue_xjal();

self.forceStatisticsCollection = self._forceStatisticsCollection_DefaultValue_xjal();

}

/**

* 设置嵌入对象实例<br>

* 这个方法不应该被用户调用

*/

@AnyLogicInternalCodegenAPI

private void create_Distributor0_xjal( com.anylogic.libraries.processmodeling.Service<Agent> self, TableInput _t ) {

self.create();

}

/**

* 创建嵌入对象实例<br>

* <i>这个方法不应该被用户调用</i>

*/

protected com.anylogic.libraries.processmodeling.Service<Agent> instantiate_Retailer0_xjal() {

com.anylogic.libraries.processmodeling.Service<Agent> _result_xjal = new com.anylogic.libraries.processmodeling.Service<Agent>( getEngine(), this, null ) {

@Override

public double delayTime( Agent agent ) {

return _Retailer0_delayTime_xjal( this, agent );

}

@AnyLogicInternalCodegenAPI

public TimeUnits getUnitsForCodeOf_delayTime() {

return DAY;

}

/**

* 这个数字在这里是出于模型快照存储的目的。它不应该被用户修改。

*/

@AnyLogicInternalCodegenAPI

private static final long serialVersionUID = -6773233648279370147L;

};

return _result_xjal;

}

/**

* 设置嵌入对象实例的参数<br>

* 这个方法不应该被用户调用

*/

private void setupParameters_Retailer0_xjal( final com.anylogic.libraries.processmodeling.Service<Agent> self ) {

setupParameters_Retailer0_xjal( self, null );

}

/**

* 设置嵌入对象实例<br>

* 这个方法不应该被用户调用

*/

@AnyLogicInternalCodegenAPI

private void create_Retailer0_xjal( com.anylogic.libraries.processmodeling.Service<Agent> self ) {

create_Retailer0_xjal(self, null );

}

/**

* 设置嵌入对象实例的参数<br>

* 这个方法不应该被用户调用

*/

private void setupParameters_Retailer0_xjal( final com.anylogic.libraries.processmodeling.Service<Agent> self, TableInput _t ) {

self.seizeFromOnePool = self._seizeFromOnePool_DefaultValue_xjal();

self.queueCapacity = self._queueCapacity_DefaultValue_xjal();

self.maximumCapacity =

true

;

self.destinationType = self._destinationType_DefaultValue_xjal();

self.entityLocationQueue = self._entityLocationQueue_DefaultValue_xjal();

self.entityLocationDelay = self._entityLocationDelay_DefaultValue_xjal();

self.suspendResumeEntities = self._suspendResumeEntities_DefaultValue_xjal();

self.customizeResourceChoice = self._customizeResourceChoice_DefaultValue_xjal();

self.enableTimeout = self._enableTimeout_DefaultValue_xjal();

self.enablePreemption = self._enablePreemption_DefaultValue_xjal();

self.restoreEntityLocationOnExit = self._restoreEntityLocationOnExit_DefaultValue_xjal();

self.forceStatisticsCollection = self._forceStatisticsCollection_DefaultValue_xjal();

}

/**

* 设置嵌入对象实例<br>

* 这个方法不应该被用户调用

*/

@AnyLogicInternalCodegenAPI

private void create_Retailer0_xjal( com.anylogic.libraries.processmodeling.Service<Agent> self, TableInput _t ) {

self.create();

}

/**

* 创建嵌入对象实例<br>

* <i>这个方法不应该被用户调用</i>

*/

protected com.anylogic.libraries.processmodeling.SelectOutput<Agent> instantiate_selectOutput_xjal() {

com.anylogic.libraries.processmodeling.SelectOutput<Agent> _result_xjal = new com.anylogic.libraries.processmodeling.SelectOutput<Agent>( getEngine(), this, null ) {

@Override

public double probability( Agent agent ) {

return _selectOutput_probability_xjal( this, agent );

}

/**

* 这个数字在这里是出于模型快照存储的目的。它不应该被用户修改。

*/

@AnyLogicInternalCodegenAPI

private static final long serialVersionUID = -6773233648279697827L;

};

return _result_xjal;

}

/**

* 设置嵌入对象实例的参数<br>

* 这个方法不应该被用户调用

*/

private void setupParameters_selectOutput_xjal( final com.anylogic.libraries.processmodeling.SelectOutput<Agent> self ) {

setupParameters_selectOutput_xjal( self, null );

}

/**

* 设置嵌入对象实例<br>

* 这个方法不应该被用户调用

*/

@AnyLogicInternalCodegenAPI

private void create_selectOutput_xjal( com.anylogic.libraries.processmodeling.SelectOutput<Agent> self ) {

create_selectOutput_xjal(self, null );

}

/**

* 设置嵌入对象实例的参数<br>

* 这个方法不应该被用户调用

*/

private void setupParameters_selectOutput_xjal( final com.anylogic.libraries.processmodeling.SelectOutput<Agent> self, TableInput _t ) {

self.conditionIsProbabilistic = self._conditionIsProbabilistic_DefaultValue_xjal();

}

/**

* 设置嵌入对象实例<br>

* 这个方法不应该被用户调用

*/

@AnyLogicInternalCodegenAPI

private void create_selectOutput_xjal( com.anylogic.libraries.processmodeling.SelectOutput<Agent> self, TableInput _t ) {

self.create();

}

/**

* 创建嵌入对象实例<br>

* <i>这个方法不应该被用户调用</i>

*/

protected com.anylogic.libraries.processmodeling.Sink<Agent> instantiate_sink_xjal() {

com.anylogic.libraries.processmodeling.Sink<Agent> _result_xjal = new com.anylogic.libraries.processmodeling.Sink<Agent>( getEngine(), this, null );

return _result_xjal;

}

/**

* 设置嵌入对象实例的参数<br>

* 这个方法不应该被用户调用

*/

private void setupParameters_sink_xjal( final com.anylogic.libraries.processmodeling.Sink<Agent> self ) {

setupParameters_sink_xjal( self, null );

}

/**

* 设置嵌入对象实例<br>

* 这个方法不应该被用户调用

*/

@AnyLogicInternalCodegenAPI

private void create_sink_xjal( com.anylogic.libraries.processmodeling.Sink<Agent> self ) {

create_sink_xjal(self, null );

}

/**

* 设置嵌入对象实例的参数<br>

* 这个方法不应该被用户调用

*/

private void setupParameters_sink_xjal( final com.anylogic.libraries.processmodeling.Sink<Agent> self, TableInput _t ) {

}

/**

* 设置嵌入对象实例<br>

* 这个方法不应该被用户调用

*/

@AnyLogicInternalCodegenAPI

private void create_sink_xjal( com.anylogic.libraries.processmodeling.Sink<Agent> self, TableInput _t ) {

self.create();

}

/**

* 创建嵌入对象实例<br>

* <i>这个方法不应该被用户调用</i>

*/

protected com.anylogic.libraries.processmodeling.SelectOutput<Agent> instantiate_selectOutput1_xjal() {

com.anylogic.libraries.processmodeling.SelectOutput<Agent> _result_xjal = new com.anylogic.libraries.processmodeling.SelectOutput<Agent>( getEngine(), this, null ) {

@Override

public double probability( Agent agent ) {

return _selectOutput1_probability_xjal( this, agent );

}

/**

* 这个数字在这里是出于模型快照存储的目的。它不应该被用户修改。

*/

@AnyLogicInternalCodegenAPI

private static final long serialVersionUID = -6773233648279633395L;

};

return _result_xjal;

}

/**

* 设置嵌入对象实例的参数<br>

* 这个方法不应该被用户调用

*/

private void setupParameters_selectOutput1_xjal( final com.anylogic.libraries.processmodeling.SelectOutput<Agent> self ) {

setupParameters_selectOutput1_xjal( self, null );

}

/**

* 设置嵌入对象实例<br>

* 这个方法不应该被用户调用

*/

@AnyLogicInternalCodegenAPI

private void create_selectOutput1_xjal( com.anylogic.libraries.processmodeling.SelectOutput<Agent> self ) {

create_selectOutput1_xjal(self, null );

}

/**

* 设置嵌入对象实例的参数<br>

* 这个方法不应该被用户调用

*/

private void setupParameters_selectOutput1_xjal( final com.anylogic.libraries.processmodeling.SelectOutput<Agent> self, TableInput _t ) {

self.conditionIsProbabilistic = self._conditionIsProbabilistic_DefaultValue_xjal();

}

/**

* 设置嵌入对象实例<br>

* 这个方法不应该被用户调用

*/

@AnyLogicInternalCodegenAPI

private void create_selectOutput1_xjal( com.anylogic.libraries.processmodeling.SelectOutput<Agent> self, TableInput _t ) {

self.create();

}

/**

* 创建嵌入对象实例<br>

* <i>这个方法不应该被用户调用</i>

*/

protected com.anylogic.libraries.processmodeling.Sink<Agent> instantiate_sink1_xjal() {

com.anylogic.libraries.processmodeling.Sink<Agent> _result_xjal = new com.anylogic.libraries.processmodeling.Sink<Agent>( getEngine(), this, null );

return _result_xjal;

}

/**

* 设置嵌入对象实例的参数<br>

* 这个方法不应该被用户调用

*/

private void setupParameters_sink1_xjal( final com.anylogic.libraries.processmodeling.Sink<Agent> self ) {

setupParameters_sink1_xjal( self, null );

}

/**

* 设置嵌入对象实例<br>

* 这个方法不应该被用户调用

*/

@AnyLogicInternalCodegenAPI

private void create_sink1_xjal( com.anylogic.libraries.processmodeling.Sink<Agent> self ) {

create_sink1_xjal(self, null );

}

/**

* 设置嵌入对象实例的参数<br>

* 这个方法不应该被用户调用

*/

private void setupParameters_sink1_xjal( final com.anylogic.libraries.processmodeling.Sink<Agent> self, TableInput _t ) {

}

/**

* 设置嵌入对象实例<br>

* 这个方法不应该被用户调用

*/

@AnyLogicInternalCodegenAPI

private void create_sink1_xjal( com.anylogic.libraries.processmodeling.Sink<Agent> self, TableInput _t ) {

self.create();

}

/**

* 创建嵌入对象实例<br>

* <i>这个方法不应该被用户调用</i>

*/

protected com.anylogic.libraries.processmodeling.Sink<Agent> instantiate_sink2_xjal() {

com.anylogic.libraries.processmodeling.Sink<Agent> _result_xjal = new com.anylogic.libraries.processmodeling.Sink<Agent>( getEngine(), this, null );

return _result_xjal;

}

/**

* 设置嵌入对象实例的参数<br>

* 这个方法不应该被用户调用

*/

private void setupParameters_sink2_xjal( final com.anylogic.libraries.processmodeling.Sink<Agent> self ) {

setupParameters_sink2_xjal( self, null );

}

/**

* 设置嵌入对象实例<br>

* 这个方法不应该被用户调用

*/

@AnyLogicInternalCodegenAPI

private void create_sink2_xjal( com.anylogic.libraries.processmodeling.Sink<Agent> self ) {

create_sink2_xjal(self, null );

}

/**

* 设置嵌入对象实例的参数<br>

* 这个方法不应该被用户调用

*/

private void setupParameters_sink2_xjal( final com.anylogic.libraries.processmodeling.Sink<Agent> self, TableInput _t ) {

}

/**

* 设置嵌入对象实例<br>

* 这个方法不应该被用户调用

*/

@AnyLogicInternalCodegenAPI

private void create_sink2_xjal( com.anylogic.libraries.processmodeling.Sink<Agent> self, TableInput _t ) {

self.create();

}

/**

* 创建嵌入对象实例<br>

* <i>这个方法不应该被用户调用</i>

*/

protected com.anylogic.libraries.processmodeling.Source<Agent> instantiate_Manufacturer0_xjal() {

com.anylogic.libraries.processmodeling.Source<Agent> _result_xjal = new com.anylogic.libraries.processmodeling.Source<Agent>( getEngine(), this, null );

return _result_xjal;

}

/**

* 设置嵌入对象实例的参数<br>

* 这个方法不应该被用户调用

*/

private void setupParameters_Manufacturer0_xjal( final com.anylogic.libraries.processmodeling.Source<Agent> self ) {

setupParameters_Manufacturer0_xjal( self, null );

}

/**

* 设置嵌入对象实例<br>

* 这个方法不应该被用户调用

*/

@AnyLogicInternalCodegenAPI

private void create_Manufacturer0_xjal( com.anylogic.libraries.processmodeling.Source<Agent> self ) {

create_Manufacturer0_xjal(self, null );

}

/**

* 设置嵌入对象实例的参数<br>

* 这个方法不应该被用户调用

*/

private void setupParameters_Manufacturer0_xjal( final com.anylogic.libraries.processmodeling.Source<Agent> self, TableInput _t ) {

self.arrivalType =

self.MANUAL

;

self.rate = self._rate_DefaultValue_xjal();

self.rateSchedule = self._rateSchedule_DefaultValue_xjal();

self.modifyRate = self._modifyRate_DefaultValue_xjal();

self.arrivalSchedule = self._arrivalSchedule_DefaultValue_xjal();

self.setAgentParametersFromDB = self._setAgentParametersFromDB_DefaultValue_xjal();

self.databaseTable = self._databaseTable_DefaultValue_xjal();

self.multipleEntitiesPerArrival = self._multipleEntitiesPerArrival_DefaultValue_xjal();

self.limitArrivals = self._limitArrivals_DefaultValue_xjal();

self.maxArrivals = self._maxArrivals_DefaultValue_xjal();

self.locationType = self._locationType_DefaultValue_xjal();

self.locationXYZInNetwork = self._locationXYZInNetwork_DefaultValue_xjal();

self.enableCustomStartTime = self._enableCustomStartTime_DefaultValue_xjal();

self.startTime = self._startTime_DefaultValue_xjal();

self.addToCustomPopulation = self._addToCustomPopulation_DefaultValue_xjal();

self.pushProtocol = self._pushProtocol_DefaultValue_xjal();

self.discardHangingEntities = self._discardHangingEntities_DefaultValue_xjal();

}

/**

* 设置嵌入对象实例<br>

* 这个方法不应该被用户调用

*/

@AnyLogicInternalCodegenAPI

private void create_Manufacturer0_xjal( com.anylogic.libraries.processmodeling.Source<Agent> self, TableInput _t ) {

self.create();

}

/**

* 创建嵌入对象实例<br>

* <i>这个方法不应该被用户调用</i>

*/

protected com.anylogic.libraries.processmodeling.Source<Agent> instantiate_Manufacturer1_xjal() {

com.anylogic.libraries.processmodeling.Source<Agent> _result_xjal = new com.anylogic.libraries.processmodeling.Source<Agent>( getEngine(), this, null );

return _result_xjal;

}

/**

* 设置嵌入对象实例的参数<br>

* 这个方法不应该被用户调用

*/

private void setupParameters_Manufacturer1_xjal( final com.anylogic.libraries.processmodeling.Source<Agent> self ) {

setupParameters_Manufacturer1_xjal( self, null );

}

/**

* 设置嵌入对象实例<br>

* 这个方法不应该被用户调用

*/

@AnyLogicInternalCodegenAPI

private void create_Manufacturer1_xjal( com.anylogic.libraries.processmodeling.Source<Agent> self ) {

create_Manufacturer1_xjal(self, null );

}

/**

* 设置嵌入对象实例的参数<br>

* 这个方法不应该被用户调用

*/

private void setupParameters_Manufacturer1_xjal( final com.anylogic.libraries.processmodeling.Source<Agent> self, TableInput _t ) {

self.arrivalType =

self.MANUAL

;

self.rate = self._rate_DefaultValue_xjal();

self.rateSchedule = self._rateSchedule_DefaultValue_xjal();

self.modifyRate = self._modifyRate_DefaultValue_xjal();

self.arrivalSchedule = self._arrivalSchedule_DefaultValue_xjal();

self.setAgentParametersFromDB = self._setAgentParametersFromDB_DefaultValue_xjal();

self.databaseTable = self._databaseTable_DefaultValue_xjal();

self.multipleEntitiesPerArrival = self._multipleEntitiesPerArrival_DefaultValue_xjal();

self.limitArrivals = self._limitArrivals_DefaultValue_xjal();

self.maxArrivals = self._maxArrivals_DefaultValue_xjal();

self.locationType = self._locationType_DefaultValue_xjal();

self.locationXYZInNetwork = self._locationXYZInNetwork_DefaultValue_xjal();

self.enableCustomStartTime = self._enableCustomStartTime_DefaultValue_xjal();

self.startTime = self._startTime_DefaultValue_xjal();

self.addToCustomPopulation = self._addToCustomPopulation_DefaultValue_xjal();

self.pushProtocol = self._pushProtocol_DefaultValue_xjal();

self.discardHangingEntities = self._discardHangingEntities_DefaultValue_xjal();

}

/**

* 设置嵌入对象实例<br>

* 这个方法不应该被用户调用

*/

@AnyLogicInternalCodegenAPI

private void create_Manufacturer1_xjal( com.anylogic.libraries.processmodeling.Source<Agent> self, TableInput _t ) {

self.create();

}

/**

* 创建嵌入对象实例<br>

* <i>这个方法不应该被用户调用</i>

*/

protected com.anylogic.libraries.processmodeling.Hold<Agent> instantiate_hold_xjal() {

com.anylogic.libraries.processmodeling.Hold<Agent> _result_xjal = new com.anylogic.libraries.processmodeling.Hold<Agent>( getEngine(), this, null );

return _result_xjal;

}

/**

* 设置嵌入对象实例的参数<br>

* 这个方法不应该被用户调用

*/

private void setupParameters_hold_xjal( final com.anylogic.libraries.processmodeling.Hold<Agent> self ) {

setupParameters_hold_xjal( self, null );

}

/**

* 设置嵌入对象实例<br>

* 这个方法不应该被用户调用

*/

@AnyLogicInternalCodegenAPI

private void create_hold_xjal( com.anylogic.libraries.processmodeling.Hold<Agent> self ) {

create_hold_xjal(self, null );

}

/**

* 设置嵌入对象实例的参数<br>

* 这个方法不应该被用户调用

*/

private void setupParameters_hold_xjal( final com.anylogic.libraries.processmodeling.Hold<Agent> self, TableInput _t ) {

self.mode = self._mode_DefaultValue_xjal();

self.nEntitiesForSelfBlock = self._nEntitiesForSelfBlock_DefaultValue_xjal();

self.initiallyBlocked =

true

;

}

/**

* 设置嵌入对象实例<br>

* 这个方法不应该被用户调用

*/

@AnyLogicInternalCodegenAPI

private void create_hold_xjal( com.anylogic.libraries.processmodeling.Hold<Agent> self, TableInput _t ) {

self.create();

}

/**

* 创建嵌入对象实例<br>

* <i>这个方法不应该被用户调用</i>

*/

protected com.anylogic.libraries.processmodeling.Hold<Agent> instantiate_hold1_xjal() {

com.anylogic.libraries.processmodeling.Hold<Agent> _result_xjal = new com.anylogic.libraries.processmodeling.Hold<Agent>( getEngine(), this, null );

return _result_xjal;

}

/**

* 设置嵌入对象实例的参数<br>

* 这个方法不应该被用户调用

*/

private void setupParameters_hold1_xjal( final com.anylogic.libraries.processmodeling.Hold<Agent> self ) {

setupParameters_hold1_xjal( self, null );

}

/**

* 设置嵌入对象实例<br>

* 这个方法不应该被用户调用

*/

@AnyLogicInternalCodegenAPI

private void create_hold1_xjal( com.anylogic.libraries.processmodeling.Hold<Agent> self ) {

create_hold1_xjal(self, null );

}

/**

* 设置嵌入对象实例的参数<br>

* 这个方法不应该被用户调用

*/

private void setupParameters_hold1_xjal( final com.anylogic.libraries.processmodeling.Hold<Agent> self, TableInput _t ) {

self.mode = self._mode_DefaultValue_xjal();

self.nEntitiesForSelfBlock = self._nEntitiesForSelfBlock_DefaultValue_xjal();

self.initiallyBlocked =

true

;

}

/**

* 设置嵌入对象实例<br>

* 这个方法不应该被用户调用

*/

@AnyLogicInternalCodegenAPI

private void create_hold1_xjal( com.anylogic.libraries.processmodeling.Hold<Agent> self, TableInput _t ) {

self.create();

}

/**

* 创建嵌入对象实例<br>

* <i>这个方法不应该被用户调用</i>

*/

protected com.anylogic.libraries.processmodeling.Delay<Agent> instantiate_delay1_xjal() {

com.anylogic.libraries.processmodeling.Delay<Agent> _result_xjal = new com.anylogic.libraries.processmodeling.Delay<Agent>( getEngine(), this, null );

return _result_xjal;

}

/**

* 设置嵌入对象实例的参数<br>

* 这个方法不应该被用户调用

*/

private void setupParameters_delay1_xjal( final com.anylogic.libraries.processmodeling.Delay<Agent> self ) {

setupParameters_delay1_xjal( self, null );

}

/**

* 设置嵌入对象实例<br>

* 这个方法不应该被用户调用

*/

@AnyLogicInternalCodegenAPI

private void create_delay1_xjal( com.anylogic.libraries.processmodeling.Delay<Agent> self ) {

create_delay1_xjal(self, null );

}

/**

* 设置嵌入对象实例的参数<br>

* 这个方法不应该被用户调用

*/

private void setupParameters_delay1_xjal( final com.anylogic.libraries.processmodeling.Delay<Agent> self, TableInput _t ) {

self.type =

self.MANUAL

;

self.capacity =

50

;

self.maximumCapacity = self._maximumCapacity_DefaultValue_xjal();

self.entityLocation = self._entityLocation_DefaultValue_xjal();

self.pushProtocol = self._pushProtocol_DefaultValue_xjal();

self.restoreEntityLocationOnExit = self._restoreEntityLocationOnExit_DefaultValue_xjal();

self.forceStatisticsCollection = self._forceStatisticsCollection_DefaultValue_xjal();

}

/**

* 设置嵌入对象实例<br>

* 这个方法不应该被用户调用

*/

@AnyLogicInternalCodegenAPI

private void create_delay1_xjal( com.anylogic.libraries.processmodeling.Delay<Agent> self, TableInput _t ) {

self.create();

}

/**

* 创建嵌入对象实例<br>

* <i>这个方法不应该被用户调用</i>

*/

protected com.anylogic.libraries.processmodeling.Delay<Agent> instantiate_delay_xjal() {

com.anylogic.libraries.processmodeling.Delay<Agent> _result_xjal = new com.anylogic.libraries.processmodeling.Delay<Agent>( getEngine(), this, null );

return _result_xjal;

}

/**

* 设置嵌入对象实例的参数<br>

* 这个方法不应该被用户调用

*/

private void setupParameters_delay_xjal( final com.anylogic.libraries.processmodeling.Delay<Agent> self ) {

setupParameters_delay_xjal( self, null );

}

/**

* 设置嵌入对象实例<br>

* 这个方法不应该被用户调用

*/

@AnyLogicInternalCodegenAPI

private void create_delay_xjal( com.anylogic.libraries.processmodeling.Delay<Agent> self ) {

create_delay_xjal(self, null );

}

/**

* 设置嵌入对象实例的参数<br>

* 这个方法不应该被用户调用

*/

private void setupParameters_delay_xjal( final com.anylogic.libraries.processmodeling.Delay<Agent> self, TableInput _t ) {

self.type =

self.MANUAL

;

self.capacity =

50

;

self.maximumCapacity = self._maximumCapacity_DefaultValue_xjal();

self.entityLocation = self._entityLocation_DefaultValue_xjal();

self.pushProtocol = self._pushProtocol_DefaultValue_xjal();

self.restoreEntityLocationOnExit = self._restoreEntityLocationOnExit_DefaultValue_xjal();

self.forceStatisticsCollection = self._forceStatisticsCollection_DefaultValue_xjal();

}

/**

* 设置嵌入对象实例<br>

* 这个方法不应该被用户调用

*/

@AnyLogicInternalCodegenAPI

private void create_delay_xjal( com.anylogic.libraries.processmodeling.Delay<Agent> self, TableInput _t ) {

self.create();

}

/**

* 创建嵌入对象实例<br>

* <i>这个方法不应该被用户调用</i>

*/

protected com.anylogic.libraries.processmodeling.Delay<Agent> instantiate_delay2_xjal() {

com.anylogic.libraries.processmodeling.Delay<Agent> _result_xjal = new com.anylogic.libraries.processmodeling.Delay<Agent>( getEngine(), this, null ) {

@Override

public double delayTime( Agent agent ) {

return _delay2_delayTime_xjal( this, agent );

}

@AnyLogicInternalCodegenAPI

public TimeUnits getUnitsForCodeOf_delayTime() {

return MONTH;

}

/**

* 这个数字在这里是出于模型快照存储的目的。它不应该被用户修改。

*/

@AnyLogicInternalCodegenAPI

private static final long serialVersionUID = -6773233642911788280L;

};

return _result_xjal;

}

/**

* 设置嵌入对象实例的参数<br>

* 这个方法不应该被用户调用

*/

private void setupParameters_delay2_xjal( final com.anylogic.libraries.processmodeling.Delay<Agent> self ) {

setupParameters_delay2_xjal( self, null );

}

/**

* 设置嵌入对象实例<br>

* 这个方法不应该被用户调用

*/

@AnyLogicInternalCodegenAPI

private void create_delay2_xjal( com.anylogic.libraries.processmodeling.Delay<Agent> self ) {

create_delay2_xjal(self, null );

}

/**

* 设置嵌入对象实例的参数<br>

* 这个方法不应该被用户调用

*/

private void setupParameters_delay2_xjal( final com.anylogic.libraries.processmodeling.Delay<Agent> self, TableInput _t ) {

self.type = self._type_DefaultValue_xjal();

self.capacity = self._capacity_DefaultValue_xjal();

self.maximumCapacity =

true

;

self.entityLocation = self._entityLocation_DefaultValue_xjal();

self.pushProtocol = self._pushProtocol_DefaultValue_xjal();

self.restoreEntityLocationOnExit = self._restoreEntityLocationOnExit_DefaultValue_xjal();

self.forceStatisticsCollection = self._forceStatisticsCollection_DefaultValue_xjal();

}

/**

* 设置嵌入对象实例<br>

* 这个方法不应该被用户调用

*/

@AnyLogicInternalCodegenAPI

private void create_delay2_xjal( com.anylogic.libraries.processmodeling.Delay<Agent> self, TableInput _t ) {

self.create();

}

private com.anylogic.libraries.processmodeling.ResourcePool[][] _Supplier0_resourceSets_xjal( final com.anylogic.libraries.processmodeling.Service<Agent> self, Agent agent ) {

com.anylogic.libraries.processmodeling.ResourcePool[][] _value;

_value = new com.anylogic.libraries.processmodeling.ResourcePool[][]

{

{ }

}

;

return _value;

}

private double _Supplier0_delayTime_xjal( final com.anylogic.libraries.processmodeling.Service<Agent> self, Agent agent ) {

double _value;

_value =

0

;

_value = DAY.convertTo( _value, SECOND );

return _value;

}

private double _Distributor0_delayTime_xjal( final com.anylogic.libraries.processmodeling.Service<Agent> self, Agent agent ) {

double _value;

_value =

0

;

_value = DAY.convertTo( _value, SECOND );

return _value;

}

private double _Retailer0_delayTime_xjal( final com.anylogic.libraries.processmodeling.Service<Agent> self, Agent agent ) {

double _value;

_value =

10

;

_value = DAY.convertTo( _value, SECOND );

return _value;

}

private double _selectOutput_probability_xjal( final com.anylogic.libraries.processmodeling.SelectOutput<Agent> self, Agent agent ) {

double _value;

_value =

u

;

return _value;

}

private double _selectOutput1_probability_xjal( final com.anylogic.libraries.processmodeling.SelectOutput<Agent> self, Agent agent ) {

double _value;

_value =

u

;

return _value;

}

private double _delay2_delayTime_xjal( final com.anylogic.libraries.processmodeling.Delay<Agent> self, Agent agent ) {

double _value;

_value =

1

;

_value = MONTH.convertTo( _value, SECOND );

return _value;

}

@AnyLogicInternalCodegenAPI

public DataSet _plot_expression0_dataSet_xjal = new DataSet( 10000000, new DataUpdater_xjal() {

double _lastUpdateX = Double.NaN;

@Override

public void update( DataSet _d ) {

if ( time() == _lastUpdateX ) { return; }

_d.add( time(), __plot_expression0_dataSet_xjal_YValue() );

_lastUpdateX = time();

}

/**

* 这个数字在这里是出于模型快照存储的目的。它不应该被用户修改。

*/

@AnyLogicInternalCodegenAPI

private static final long serialVersionUID = -6773233648280418739L;

} );

/**

* <i>这个方法不应该被用户调用</i>

*/

@AnyLogicInternalCodegenAPI

private double __plot_expression0_dataSet_xjal_YValue() {

return

Supplier0.size()

;

}

@AnyLogicInternalCodegenAPI

public DataSet _plot1_expression0_dataSet_xjal = new DataSet( 10000000, new DataUpdater_xjal() {

double _lastUpdateX = Double.NaN;

@Override

public void update( DataSet _d ) {

if ( time() == _lastUpdateX ) { return; }

_d.add( time(), __plot1_expression0_dataSet_xjal_YValue() );

_lastUpdateX = time();

}

/**

* 这个数字在这里是出于模型快照存储的目的。它不应该被用户修改。

*/

@AnyLogicInternalCodegenAPI

private static final long serialVersionUID = -6773233648280418739L;

} );

/**

* <i>这个方法不应该被用户调用</i>

*/

@AnyLogicInternalCodegenAPI

private double __plot1_expression0_dataSet_xjal_YValue() {

return

Distributor0.size()

;

}

@AnyLogicInternalCodegenAPI

public DataSet _plot3_expression0_dataSet_xjal = new DataSet( 100, new DataUpdater_xjal() {

double _lastUpdateX = Double.NaN;

@Override

public void update( DataSet _d ) {

if ( time() == _lastUpdateX ) { return; }

_d.add( time(), __plot3_expression0_dataSet_xjal_YValue() );

_lastUpdateX = time();

}

/**

* 这个数字在这里是出于模型快照存储的目的。它不应该被用户修改。

*/

@AnyLogicInternalCodegenAPI

private static final long serialVersionUID = -6773233648280418739L;

} );

/**

* <i>这个方法不应该被用户调用</i>

*/

@AnyLogicInternalCodegenAPI

private double __plot3_expression0_dataSet_xjal_YValue() {

return

p1*Manufacturer0.out.count()*30+sink.in.count()*l+p1*Manufacturer1.out.count()*30+l*sink1.in.count()

;

}//calculate total cost

@AnyLogicInternalCodegenAPI

public DataSet _plot2_expression0_dataSet_xjal = new DataSet( 100, new DataUpdater_xjal() {

double _lastUpdateX = Double.NaN;

@Override

public void update( DataSet _d ) {

if ( time() == _lastUpdateX ) { return; }

_d.add( time(), __plot2_expression0_dataSet_xjal_YValue() );

_lastUpdateX = time();

}

/**

* 这个数字在这里是出于模型快照存储的目的。它不应该被用户修改。

*/

@AnyLogicInternalCodegenAPI

private static final long serialVersionUID = -6773233648280418739L;

} );

/**

* <i>这个方法不应该被用户调用</i>

*/

@AnyLogicInternalCodegenAPI

private double __plot2_expression0_dataSet_xjal_YValue() {

return

Manufacturer1.out.count()

;

}

@AnyLogicInternalCodegenAPI

public DataSet _plot4_expression0_dataSet_xjal = new DataSet( 100, new DataUpdater_xjal() {

double _lastUpdateX = Double.NaN;

@Override

public void update( DataSet _d ) {

if ( time() == _lastUpdateX ) { return; }

_d.add( time(), __plot4_expression0_dataSet_xjal_YValue() );

_lastUpdateX = time();

}

/**

* 这个数字在这里是出于模型快照存储的目的。它不应该被用户修改。

*/

@AnyLogicInternalCodegenAPI

private static final long serialVersionUID = -6773233648280418739L;

} );

/**

* <i>这个方法不应该被用户调用</i>

*/

@AnyLogicInternalCodegenAPI

private double __plot4_expression0_dataSet_xjal_YValue() {

return

0

;

}

// 视图区域

public ViewArea viewFlow = new ViewArea( this, "Flow", 0, 0, ViewArea.TOP_LEFT, ViewArea.NONE, 1.0, 100, 100 );

public ViewArea viewAnalysisOfQuantity = new ViewArea( this, "Analysis", 0, 600, ViewArea.TOP_LEFT, ViewArea.NONE, 1.0, 100, 100 );

public ViewArea viewCost = new ViewArea( this, null, 0, 1200, ViewArea.TOP_LEFT, ViewArea.NONE, 1.0, 100, 100 );

public ViewArea viewCostandQuantity = new ViewArea( this, null, 0, 1810, ViewArea.TOP_LEFT, ViewArea.NONE, 1.0, 100, 100 );

public ViewArea _origin_VA = new ViewArea( this, "[原点]", 0, 0, ViewArea.TOP_LEFT, ViewArea.SPECIFIED_ZOOM, 1, 100, 100 );

@Override

@AnyLogicInternalCodegenAPI

public int getViewAreas(Map<String, ViewArea> _output) {

if ( _output != null ) {

_output.put( "viewFlow", this.viewFlow );

_output.put( "viewAnalysisOfQuantity", this.viewAnalysisOfQuantity );

_output.put( "viewCost", this.viewCost );

_output.put( "viewCostandQuantity", this.viewCostandQuantity );

_output.put( "_origin_VA", this._origin_VA );

}

return 5 + super.getViewAreas( _output );

}

@AnyLogicInternalCodegenAPI

protected static final Font _text_Font = new Font("SansSerif", 0, 16 );

@AnyLogicInternalCodegenAPI

protected static final Font _text1_Font = _text_Font;

@AnyLogicInternalCodegenAPI

protected static final Font _text2_Font = _text_Font;

@AnyLogicInternalCodegenAPI

protected static final Font _text3_Font = _text_Font;

@AnyLogicInternalCodegenAPI

protected static final Font _text8_Font = _text_Font;

@AnyLogicInternalCodegenAPI

protected static final Font _text6_Font = _text_Font;

@AnyLogicInternalCodegenAPI

protected static final Font _text9_Font = _text_Font;

@AnyLogicInternalCodegenAPI

protected static final Font _text10_Font = _text_Font;

@AnyLogicInternalCodegenAPI

protected static final Font _text11_Font = _text_Font;

@AnyLogicInternalCodegenAPI

protected static final Font _text12_Font = _text_Font;

@AnyLogicInternalCodegenAPI

protected static final Font _text14_Font = _text_Font;

@AnyLogicInternalCodegenAPI

protected static final Font _text15_Font = _text_Font;

@AnyLogicInternalCodegenAPI

protected static final Font _text5_Font = _text_Font;

@AnyLogicInternalCodegenAPI

protected static final Font _text4_Font = _text_Font;

@AnyLogicInternalCodegenAPI

protected static final Font _text7_Font = _text_Font;

@AnyLogicInternalCodegenAPI

protected static final Font _text13_Font = _text_Font;

@AnyLogicInternalCodegenAPI

protected static final int _slider2 = 1;

@AnyLogicInternalCodegenAPI

protected static final int _slider = 2;

@AnyLogicInternalCodegenAPI

protected static final int _text = 3;

@AnyLogicInternalCodegenAPI

protected static final int _text1 = 4;

@AnyLogicInternalCodegenAPI

protected static final int _line = 5;

@AnyLogicInternalCodegenAPI

protected static final int _text2 = 6;

@AnyLogicInternalCodegenAPI

protected static final int _line1 = 7;

@AnyLogicInternalCodegenAPI

protected static final int _text3 = 8;

@AnyLogicInternalCodegenAPI

protected static final int _text8 = 9;

@AnyLogicInternalCodegenAPI

protected static final int _line4 = 10;

@AnyLogicInternalCodegenAPI

protected static final int _text6 = 11;

@AnyLogicInternalCodegenAPI

protected static final int _line5 = 12;

@AnyLogicInternalCodegenAPI

protected static final int _text9 = 13;

@AnyLogicInternalCodegenAPI

protected static final int _line6 = 14;

@AnyLogicInternalCodegenAPI

protected static final int _text10 = 15;

@AnyLogicInternalCodegenAPI

protected static final int _line7 = 16;

@AnyLogicInternalCodegenAPI

protected static final int _text11 = 17;

@AnyLogicInternalCodegenAPI

protected static final int _line8 = 18;

@AnyLogicInternalCodegenAPI

protected static final int _text12 = 19;

@AnyLogicInternalCodegenAPI

protected static final int _text14 = 20;

@AnyLogicInternalCodegenAPI

protected static final int _line10 = 21;

@AnyLogicInternalCodegenAPI

protected static final int _text15 = 22;

@AnyLogicInternalCodegenAPI

protected static final int _line11 = 23;

@AnyLogicInternalCodegenAPI

protected static final int _text5 = 24;

@AnyLogicInternalCodegenAPI

protected static final int _line2 = 25;

@AnyLogicInternalCodegenAPI

protected static final int _text4 = 26;

@AnyLogicInternalCodegenAPI

protected static final int _line3 = 27;

@AnyLogicInternalCodegenAPI

protected static final int _text7 = 28;

@AnyLogicInternalCodegenAPI

protected static final int _text13 = 29;

@AnyLogicInternalCodegenAPI

protected static final int _line9 = 30;

@AnyLogicInternalCodegenAPI

protected static final int _plot = 31;

@AnyLogicInternalCodegenAPI

protected static final int _plot1 = 32;

@AnyLogicInternalCodegenAPI

protected static final int _plot3 = 33;

@AnyLogicInternalCodegenAPI

protected static final int _plot2 = 34;

@AnyLogicInternalCodegenAPI

protected static final int _plot4 = 35;

/** Internal constant, shouldn't be accessed by user */

@AnyLogicInternalCodegenAPI

protected static final int _SHAPE_NEXT_ID_xjal = 36;

/**

* 最高层演示组编号

*/

@AnyLogicInternalCodegenAPI

protected static final int _presentation = 0;

@AnyLogicInternalCodegenAPI

public boolean isPublicPresentationDefined() {

return true;

}

@AnyLogicInternalCodegenAPI

public boolean isEmbeddedAgentPresentationVisible( Agent _a ) {

return super.isEmbeddedAgentPresentationVisible( _a );

}

/**

* 最高层图标组编号

*/

@AnyLogicInternalCodegenAPI

protected static final int _icon = -1;

@Override

@AnyLogicInternalCodegenAPI

public boolean onShapeClick( int _shape, int index, double clickx, double clicky ){

switch( _shape ){

case _text1:

if (true) {

ShapeText self = this.text1;

viewAnalysisOfQuantity.navigateTo();

}

break;

case _text2:

if (true) {

ShapeText self = this.text2;

viewFlow.navigateTo();

}

break;

case _text8:

if (true) {

ShapeText self = this.text8;

viewCost.navigateTo();

}

break;

case _text6:

if (true) {

ShapeText self = this.text6;

viewCost.navigateTo();

}

break;

case _text9:

if (true) {

ShapeText self = this.text9;

viewAnalysisOfQuantity.navigateTo();

}

break;

case _text10:

if (true) {

ShapeText self = this.text10;

viewFlow.navigateTo();

}

break;

case _text11:

if (true) {

ShapeText self = this.text11;

viewCost.navigateTo();

}

break;

case _text14:

if (true) {

ShapeText self = this.text14;

viewCostandQuantity.navigateTo();

}

break;

case _text15:

if (true) {

ShapeText self = this.text15;

viewCostandQuantity.navigateTo();

}

break;

case _text5:

if (true) {

ShapeText self = this.text5;

viewAnalysisOfQuantity.navigateTo();

}

break;

case _text4:

if (true) {

ShapeText self = this.text4;

viewFlow.navigateTo();

}

break;

case _text13:

if (true) {

ShapeText self = this.text13;

viewCostandQuantity.navigateTo();

}

break;

default: return super.onShapeClick( _shape, index, clickx, clicky );

}

return false;

}

@Override

@AnyLogicInternalCodegenAPI

public void executeShapeControlAction( int _shape, int index, double value ) {

switch( _shape ) {

case _slider2:

set_p1( value );

break;

case _slider:

set_l( value );

break;

default:

super.executeShapeControlAction( _shape, index, value );

break;

}

}

@Override

@AnyLogicInternalCodegenAPI

public double getShapeControlMinimum( int _shape, int index ) {

switch( _shape ) {

case _slider2: return

0.2

;

case _slider: return

0

;

default: return super.getShapeControlMinimum( _shape, index );

}

}

@Override

@AnyLogicInternalCodegenAPI

public double getShapeControlMaximum( int _shape, int index ) {

switch( _shape ) {

case _slider2: return

0.5

;

case _slider: return

200

;

default: return super.getShapeControlMaximum( _shape, index );

}

}

@Override

@AnyLogicInternalCodegenAPI

public double getShapeControlDefaultValueDouble( int _shape, int index ) {

switch(_shape) {

case _slider2: return

p1

;

case _slider: return

l

;

default: return super.getShapeControlDefaultValueDouble( _shape, index );

}

}

/**

* <i>这个方法不应该被用户调用</i>

*/

@AnyLogicInternalCodegenAPI

private void _slider2_SetDynamicParams_xjal( ShapeSlider shape ) {

shape.setRange( getShapeControlMinimum( _slider2 ), getShapeControlMaximum( _slider2 ) );

}

protected ShapeSlider slider2;

/**

* <i>这个方法不应该被用户调用</i>

*/

@AnyLogicInternalCodegenAPI

private void _slider_SetDynamicParams_xjal( ShapeSlider shape ) {

shape.setRange( getShapeControlMinimum( _slider ), getShapeControlMaximum( _slider ) );

}

protected ShapeSlider slider;

protected TimePlot plot;

protected TimePlot plot1;

protected TimePlot plot3;

protected TimePlot plot2;

protected TimePlot plot4;

protected ShapeText text;

protected ShapeText text1;

protected ShapeLine line;

protected ShapeText text2;

protected ShapeLine line1;

protected ShapeText text3;

protected ShapeText text8;

protected ShapeLine line4;

protected ShapeText text6;

protected ShapeLine line5;

protected ShapeText text9;

protected ShapeLine line6;

protected ShapeText text10;

protected ShapeLine line7;

protected ShapeText text11;

protected ShapeLine line8;

protected ShapeText text12;

protected ShapeText text14;

protected ShapeLine line10;

protected ShapeText text15;

protected ShapeLine line11;

protected ShapeText text5;

protected ShapeLine line2;

protected ShapeText text4;

protected ShapeLine line3;

protected ShapeText text7;

protected ShapeText text13;

protected ShapeLine line9;

@AnyLogicInternalCodegenAPI

private void _createPersistentElementsBP0_xjal() {

slider2 = new ShapeSlider(

Main.this, true, 260.0, 1000.0,

100.0, 30.0,

transparent, true,

false, getShapeControlMinimum( _slider2 ), getShapeControlMaximum( _slider2 ), ShapeControl.TYPE_DOUBLE ) {

@Override

public void updateDynamicProperties(boolean publicOnly) {

_slider2_SetDynamicParams_xjal( this );

super.updateDynamicProperties(publicOnly);

}

@Override

@AnyLogicInternalCodegenAPI

public void action(){

executeShapeControlAction( _slider2, 0, value );

}

@Override

public void setValueToDefault() {

setValue( limit( getMin(), getShapeControlDefaultValueDouble( _slider2, 0 ), getMax() ) );

}

/**

* 这个数字在这里是出于模型快照存储的目的。它不应该被用户修改。

*/

@AnyLogicInternalCodegenAPI

private static final long serialVersionUID = -6773233647286626548L;

};

slider = new ShapeSlider(

Main.this, true, 510.0, 1010.0,

100.0, 30.0,

transparent, true,

false, getShapeControlMinimum( _slider ), getShapeControlMaximum( _slider ), ShapeControl.TYPE_DOUBLE ) {

@Override

public void updateDynamicProperties(boolean publicOnly) {

_slider_SetDynamicParams_xjal( this );

super.updateDynamicProperties(publicOnly);

}

@Override

@AnyLogicInternalCodegenAPI

public void action(){

executeShapeControlAction( _slider, 0, value );

}

@Override

public void setValueToDefault() {

setValue( limit( getMin(), getShapeControlDefaultValueDouble( _slider, 0 ), getMax() ) );

}

/**

* 这个数字在这里是出于模型快照存储的目的。它不应该被用户修改。

*/

@AnyLogicInternalCodegenAPI

private static final long serialVersionUID = -6773233644001438196L;

};

text = new ShapeText(

SHAPE_DRAW_2D, true,180.0, 30.0, 0.0, 0.0,

black,"Flow",

_text_Font, ALIGNMENT_LEFT );

text1 = new ShapeText(

SHAPE_DRAW_2D, true,260.0, 30.0, 0.0, 0.0,

blue,"Analysis of Quantity",

_text1_Font, ALIGNMENT_LEFT ) {

@Override

@AnyLogicInternalCodegenAPI

public boolean onClick( double clickx, double clicky ) {

return onShapeClick( _text1, 0, clickx, clicky );

}

/**

* 这个数字在这里是出于模型快照存储的目的。它不应该被用户修改。

*/

@AnyLogicInternalCodegenAPI

private static final long serialVersionUID = -6773233648024841639L;

};

line = new ShapeLine(

SHAPE_DRAW_2D3D, true, 260.0, 50.0, 0.0, blue,

130.0, 0.0, 0.0, 1.0, 10.0, LINE_STYLE_SOLID );

text2 = new ShapeText(

SHAPE_DRAW_2D, true,180.0, 630.0, 0.0, 0.0,

blue,"Flow",

_text2_Font, ALIGNMENT_LEFT ) {

@Override

@AnyLogicInternalCodegenAPI

public boolean onClick( double clickx, double clicky ) {

return onShapeClick( _text2, 0, clickx, clicky );

}

/**

* 这个数字在这里是出于模型快照存储的目的。它不应该被用户修改。

*/

@AnyLogicInternalCodegenAPI

private static final long serialVersionUID = -6773233648024644792L;

};

line1 = new ShapeLine(

SHAPE_DRAW_2D3D, true, 180.0, 650.0, 0.0, blue,

30.0, 0.0, 0.0, 1.0, 10.0, LINE_STYLE_SOLID );

text3 = new ShapeText(

SHAPE_DRAW_2D, true,260.0, 630.0, 0.0, 0.0,

black,"Analysis of Quantity",

_text3_Font, ALIGNMENT_LEFT );

text8 = new ShapeText(

SHAPE_DRAW_2D, true,440.0, 30.0, 0.0, 0.0,

blue,"Cost",

_text8_Font, ALIGNMENT_LEFT ) {

@Override

@AnyLogicInternalCodegenAPI

public boolean onClick( double clickx, double clicky ) {

return onShapeClick( _text8, 0, clickx, clicky );

}

/**

* 这个数字在这里是出于模型快照存储的目的。它不应该被用户修改。

*/

@AnyLogicInternalCodegenAPI

private static final long serialVersionUID = -6773233647222728948L;

};

line4 = new ShapeLine(

SHAPE_DRAW_2D3D, true, 440.0, 50.0, 0.0, blue,

30.0, 0.0, 0.0, 1.0, 10.0, LINE_STYLE_SOLID );

text6 = new ShapeText(

SHAPE_DRAW_2D, true,440.0, 630.0, 0.0, 0.0,

blue,"Cost",

_text6_Font, ALIGNMENT_LEFT ) {

@Override

@AnyLogicInternalCodegenAPI

public boolean onClick( double clickx, double clicky ) {

return onShapeClick( _text6, 0, clickx, clicky );

}

/**

* 这个数字在这里是出于模型快照存储的目的。它不应该被用户修改。

*/

@AnyLogicInternalCodegenAPI

private static final long serialVersionUID = -6773233647222667763L;

};

line5 = new ShapeLine(

SHAPE_DRAW_2D3D, true, 440.0, 650.0, 0.0, blue,

30.0, 0.0, 0.0, 1.0, 10.0, LINE_STYLE_SOLID );

text9 = new ShapeText(

SHAPE_DRAW_2D, true,260.0, 1830.0, 0.0, 0.0,

blue,"Analysis of Quantity",

_text9_Font, ALIGNMENT_LEFT ) {

@Override

@AnyLogicInternalCodegenAPI

public boolean onClick( double clickx, double clicky ) {

return onShapeClick( _text9, 0, clickx, clicky );

}

/**

* 这个数字在这里是出于模型快照存储的目的。它不应该被用户修改。

*/

@AnyLogicInternalCodegenAPI

private static final long serialVersionUID = -6773233647205973476L;

};

line6 = new ShapeLine(

SHAPE_DRAW_2D3D, true, 260.0, 1850.0, 0.0, blue,

130.0, 0.0, 0.0, 1.0, 10.0, LINE_STYLE_SOLID );

text10 = new ShapeText(

SHAPE_DRAW_2D, true,190.0, 1830.0, 0.0, 0.0,

blue,"Flow",

_text10_Font, ALIGNMENT_LEFT ) {

@Override

@AnyLogicInternalCodegenAPI

public boolean onClick( double clickx, double clicky ) {

return onShapeClick( _text10, 0, clickx, clicky );

}

/**

* 这个数字在这里是出于模型快照存储的目的。它不应该被用户修改。

*/

@AnyLogicInternalCodegenAPI

private static final long serialVersionUID = -6773233647205973480L;

};

line7 = new ShapeLine(

SHAPE_DRAW_2D3D, true, 190.0, 1850.0, 0.0, blue,

30.0, 0.0, 0.0, 1.0, 10.0, LINE_STYLE_SOLID );

text11 = new ShapeText(

SHAPE_DRAW_2D, true,450.0, 1830.0, 0.0, 0.0,

blue,"Cost",

_text11_Font, ALIGNMENT_LEFT ) {

@Override

@AnyLogicInternalCodegenAPI

public boolean onClick( double clickx, double clicky ) {

return onShapeClick( _text11, 0, clickx, clicky );

}

/**

* 这个数字在这里是出于模型快照存储的目的。它不应该被用户修改。

*/

@AnyLogicInternalCodegenAPI

private static final long serialVersionUID = -6773233647205969064L;

};

line8 = new ShapeLine(

SHAPE_DRAW_2D3D, true, 450.0, 1850.0, 0.0, blue,

30.0, 0.0, 0.0, 1.0, 10.0, LINE_STYLE_SOLID );

text12 = new ShapeText(

SHAPE_DRAW_2D, true,540.0, 1830.0, 0.0, 0.0,

black,"Cost and Quantity",

_text12_Font, ALIGNMENT_LEFT );

text14 = new ShapeText(

SHAPE_DRAW_2D, true,520.0, 630.0, 0.0, 0.0,

blue,"Cost and Quantity",

_text14_Font, ALIGNMENT_LEFT ) {

@Override

@AnyLogicInternalCodegenAPI

public boolean onClick( double clickx, double clicky ) {

return onShapeClick( _text14, 0, clickx, clicky );

}

/**

* 这个数字在这里是出于模型快照存储的目的。它不应该被用户修改。

*/

@AnyLogicInternalCodegenAPI

private static final long serialVersionUID = -6773233647205891556L;

};

line10 = new ShapeLine(

SHAPE_DRAW_2D3D, true, 520.0, 650.0, 0.0, blue,

120.0, 0.0, 0.0, 1.0, 10.0, LINE_STYLE_SOLID );

text15 = new ShapeText(

SHAPE_DRAW_2D, true,530.0, 30.0, 0.0, 0.0,

blue,"Cost and Quantity",

_text15_Font, ALIGNMENT_LEFT ) {

@Override

@AnyLogicInternalCodegenAPI

public boolean onClick( double clickx, double clicky ) {

return onShapeClick( _text15, 0, clickx, clicky );

}

/**

* 这个数字在这里是出于模型快照存储的目的。它不应该被用户修改。

*/

@AnyLogicInternalCodegenAPI

private static final long serialVersionUID = -6773233647205707188L;

};

line11 = new ShapeLine(

SHAPE_DRAW_2D3D, true, 530.0, 50.0, 0.0, blue,

120.0, 0.0, 0.0, 1.0, 10.0, LINE_STYLE_SOLID );

text5 = new ShapeText(

SHAPE_DRAW_2D, true,220.0, 1230.0, 0.0, 0.0,

blue,"Analysis of Quantity",

_text5_Font, ALIGNMENT_LEFT ) {

@Override

@AnyLogicInternalCodegenAPI

public boolean onClick( double clickx, double clicky ) {

return onShapeClick( _text5, 0, clickx, clicky );

}

/**

* 这个数字在这里是出于模型快照存储的目的。它不应该被用户修改。

*/

@AnyLogicInternalCodegenAPI

private static final long serialVersionUID = -6773233647285660087L;

};

line2 = new ShapeLine(

SHAPE_DRAW_2D3D, true, 220.0, 1250.0, 0.0, blue,

130.0, 0.0, 0.0, 1.0, 10.0, LINE_STYLE_SOLID );

text4 = new ShapeText(

SHAPE_DRAW_2D, true,150.0, 1230.0, 0.0, 0.0,

blue,"Flow",

_text4_Font, ALIGNMENT_LEFT ) {

@Override

@AnyLogicInternalCodegenAPI

public boolean onClick( double clickx, double clicky ) {

return onShapeClick( _text4, 0, clickx, clicky );

}

/**

* 这个数字在这里是出于模型快照存储的目的。它不应该被用户修改。

*/

@AnyLogicInternalCodegenAPI

private static final long serialVersionUID = -6773233647285660083L;

};

line3 = new ShapeLine(

SHAPE_DRAW_2D3D, true, 150.0, 1250.0, 0.0, blue,

30.0, 0.0, 0.0, 1.0, 10.0, LINE_STYLE_SOLID );

text7 = new ShapeText(

SHAPE_DRAW_2D, true,400.0, 1230.0, 0.0, 0.0,

black,"Cost",

_text7_Font, ALIGNMENT_LEFT );

text13 = new ShapeText(

SHAPE_DRAW_2D, true,480.0, 1230.0, 0.0, 0.0,

blue,"Cost and Quantity",

_text13_Font, ALIGNMENT_LEFT ) {

@Override

@AnyLogicInternalCodegenAPI

public boolean onClick( double clickx, double clicky ) {

return onShapeClick( _text13, 0, clickx, clicky );

}

/**

* 这个数字在这里是出于模型快照存储的目的。它不应该被用户修改。

*/

@AnyLogicInternalCodegenAPI

private static final long serialVersionUID = -6773233647285660067L;

};

line9 = new ShapeLine(

SHAPE_DRAW_2D3D, true, 480.0, 1250.0, 0.0, blue,

120.0, 0.0, 0.0, 1.0, 10.0, LINE_STYLE_SOLID );

}

@AnyLogicInternalCodegenAPI

private void _createPersistentElementsAP0_xjal() {

{

DataSet _item;

List<DataSet> _items = new ArrayList<DataSet>( 1 );

_items.add( _plot_expression0_dataSet_xjal );

List<String> _titles = new ArrayList<String>( 1 );

_titles.add( "Quantity of Supplier" );

List<Chart2DPlot.Appearance> _appearances = new ArrayList<Chart2DPlot.Appearance>( 1 );

_appearances.add( new Chart2DPlot.Appearance( teal, true, Chart.INTERPOLATION_LINEAR, 2.0, Chart.POINT_NONE ) );

plot = new TimePlot(

Main.this, true, 70.0, 690.0,

260.0, 220.0,

null, null,

50.0, 30.0,

180.0, 130.0, white, black, black,

30.0, Chart.SOUTH,

100

, Chart.WINDOW_MOVES_WITH_TIME, null, Chart.SCALE_FIXED,

0

,

100

, Chart.GRID_DEFAULT, Chart.GRID_DEFAULT,

darkGray, darkGray, _items, _titles, _appearances );

}

{

DataSet _item;

List<DataSet> _items = new ArrayList<DataSet>( 1 );

_items.add( _plot1_expression0_dataSet_xjal );

List<String> _titles = new ArrayList<String>( 1 );

_titles.add( "Quantity of Distributor" );

List<Chart2DPlot.Appearance> _appearances = new ArrayList<Chart2DPlot.Appearance>( 1 );

_appearances.add( new Chart2DPlot.Appearance( peru, true, Chart.INTERPOLATION_LINEAR, 2.0, Chart.POINT_NONE ) );

plot1 = new TimePlot(

Main.this, true, 370.0, 690.0,

260.0, 220.0,

null, null,

50.0, 30.0,

180.0, 130.0, white, black, black,

30.0, Chart.SOUTH,

100

, Chart.WINDOW_MOVES_WITH_TIME, null, Chart.SCALE_AUTO,

0, 0, Chart.GRID_DEFAULT, Chart.GRID_DEFAULT,

darkGray, darkGray, _items, _titles, _appearances );

}

{

DataSet _item;

List<DataSet> _items = new ArrayList<DataSet>( 1 );

_items.add( _plot3_expression0_dataSet_xjal );

List<String> _titles = new ArrayList<String>( 1 );

_titles.add( "Dataset Title" );

List<Chart2DPlot.Appearance> _appearances = new ArrayList<Chart2DPlot.Appearance>( 1 );

_appearances.add( new Chart2DPlot.Appearance( yellowGreen, true, Chart.INTERPOLATION_LINEAR, 1.0, Chart.POINT_NONE ) );

plot3 = new TimePlot(

Main.this, true, 540.0, 1410.0,

360.0, 290.0,

null, null,

50.0, 30.0,

280.0, 200.0, white, black, black,

30.0, Chart.SOUTH,

100

, Chart.WINDOW_MOVES_WITH_TIME, null, Chart.SCALE_AUTO,

0, 0, Chart.GRID_DEFAULT, Chart.GRID_DEFAULT,

darkGray, darkGray, _items, _titles, _appearances );

}

{

DataSet _item;

List<DataSet> _items = new ArrayList<DataSet>( 1 );

_items.add( _plot2_expression0_dataSet_xjal );

List<String> _titles = new ArrayList<String>( 1 );

_titles.add( "Dataset Title" );

List<Chart2DPlot.Appearance> _appearances = new ArrayList<Chart2DPlot.Appearance>( 1 );

_appearances.add( new Chart2DPlot.Appearance( oliveDrab, true, Chart.INTERPOLATION_LINEAR, 1.0, Chart.POINT_NONE ) );

plot2 = new TimePlot(

Main.this, true, 120.0, 1950.0,

260.0, 210.0,

null, null,

50.0, 30.0,

180.0, 120.0, white, black, black,

30.0, Chart.SOUTH,

100

, Chart.WINDOW_MOVES_WITH_TIME, null, Chart.SCALE_AUTO,

0, 0, Chart.GRID_DEFAULT, Chart.GRID_DEFAULT,

darkGray, darkGray, _items, _titles, _appearances );

}

{

DataSet _item;

List<DataSet> _items = new ArrayList<DataSet>( 1 );

_items.add( _plot4_expression0_dataSet_xjal );

List<String> _titles = new ArrayList<String>( 1 );

_titles.add( "Dataset Title" );

List<Chart2DPlot.Appearance> _appearances = new ArrayList<Chart2DPlot.Appearance>( 1 );

_appearances.add( new Chart2DPlot.Appearance( slateBlue, true, Chart.INTERPOLATION_LINEAR, 1.0, Chart.POINT_NONE ) );

plot4 = new TimePlot(

Main.this, true, 420.0, 1950.0,

260.0, 210.0,

null, null,

50.0, 30.0,

180.0, 120.0, white, black, black,

30.0, Chart.SOUTH,

100

, Chart.WINDOW_MOVES_WITH_TIME, null, Chart.SCALE_AUTO,

0, 0, Chart.GRID_DEFAULT, Chart.GRID_DEFAULT,

darkGray, darkGray, _items, _titles, _appearances );

}

}

// 持久元素的静态初始化

{

_createPersistentElementsBP0_xjal();

}

protected ShapeTopLevelPresentationGroup presentation;

protected ShapeGroup icon;

@Override

@AnyLogicInternalCodegenAPI

public Object getPersistentShape( int _shape ) {

switch (_shape) {

case _presentation: return presentation;

case _icon: return icon;

case _slider2: return slider2;

case _slider: return slider;

case _plot: return plot;

case _plot1: return plot1;

case _plot3: return plot3;

case _plot2: return plot2;

case _plot4: return plot4;

case _text: return text;

case _text1: return text1;

case _line: return line;

case _text2: return text2;

case _line1: return line1;

case _text3: return text3;

case _text8: return text8;

case _line4: return line4;

case _text6: return text6;

case _line5: return line5;

case _text9: return text9;

case _line6: return line6;

case _text10: return text10;

case _line7: return line7;

case _text11: return text11;

case _line8: return line8;

case _text12: return text12;

case _text14: return text14;

case _line10: return line10;

case _text15: return text15;

case _line11: return line11;

case _text5: return text5;

case _line2: return line2;

case _text4: return text4;

case _line3: return line3;

case _text7: return text7;

case _text13: return text13;

case _line9: return line9;

default: return super.getPersistentShape( _shape );

}

}

@Override

@AnyLogicInternalCodegenAPI

public String getNameOfShape_xjal( Object _shape ) {

try {

if ( _shape == null ) return null;

String _name_xjal;

_name_xjal = checkNameOfShape_xjal( _shape, presentation, "presentation" ); if (_name_xjal != null) return _name_xjal;

_name_xjal = checkNameOfShape_xjal( _shape, icon, "icon" ); if (_name_xjal != null) return _name_xjal;

_name_xjal = checkNameOfShape_xjal( _shape, slider2, "slider2" ); if (_name_xjal != null) return _name_xjal;

_name_xjal = checkNameOfShape_xjal( _shape, slider, "slider" ); if (_name_xjal != null) return _name_xjal;

_name_xjal = checkNameOfShape_xjal( _shape, plot, "plot" ); if (_name_xjal != null) return _name_xjal;

_name_xjal = checkNameOfShape_xjal( _shape, plot1, "plot1" ); if (_name_xjal != null) return _name_xjal;

_name_xjal = checkNameOfShape_xjal( _shape, plot3, "plot3" ); if (_name_xjal != null) return _name_xjal;

_name_xjal = checkNameOfShape_xjal( _shape, plot2, "plot2" ); if (_name_xjal != null) return _name_xjal;

_name_xjal = checkNameOfShape_xjal( _shape, plot4, "plot4" ); if (_name_xjal != null) return _name_xjal;

_name_xjal = checkNameOfShape_xjal( _shape, text, "text" ); if (_name_xjal != null) return _name_xjal;

_name_xjal = checkNameOfShape_xjal( _shape, text1, "text1" ); if (_name_xjal != null) return _name_xjal;

_name_xjal = checkNameOfShape_xjal( _shape, line, "line" ); if (_name_xjal != null) return _name_xjal;

_name_xjal = checkNameOfShape_xjal( _shape, text2, "text2" ); if (_name_xjal != null) return _name_xjal;

_name_xjal = checkNameOfShape_xjal( _shape, line1, "line1" ); if (_name_xjal != null) return _name_xjal;

_name_xjal = checkNameOfShape_xjal( _shape, text3, "text3" ); if (_name_xjal != null) return _name_xjal;

_name_xjal = checkNameOfShape_xjal( _shape, text8, "text8" ); if (_name_xjal != null) return _name_xjal;

_name_xjal = checkNameOfShape_xjal( _shape, line4, "line4" ); if (_name_xjal != null) return _name_xjal;

_name_xjal = checkNameOfShape_xjal( _shape, text6, "text6" ); if (_name_xjal != null) return _name_xjal;

_name_xjal = checkNameOfShape_xjal( _shape, line5, "line5" ); if (_name_xjal != null) return _name_xjal;

_name_xjal = checkNameOfShape_xjal( _shape, text9, "text9" ); if (_name_xjal != null) return _name_xjal;

_name_xjal = checkNameOfShape_xjal( _shape, line6, "line6" ); if (_name_xjal != null) return _name_xjal;

_name_xjal = checkNameOfShape_xjal( _shape, text10, "text10" ); if (_name_xjal != null) return _name_xjal;

_name_xjal = checkNameOfShape_xjal( _shape, line7, "line7" ); if (_name_xjal != null) return _name_xjal;

_name_xjal = checkNameOfShape_xjal( _shape, text11, "text11" ); if (_name_xjal != null) return _name_xjal;

_name_xjal = checkNameOfShape_xjal( _shape, line8, "line8" ); if (_name_xjal != null) return _name_xjal;

_name_xjal = checkNameOfShape_xjal( _shape, text12, "text12" ); if (_name_xjal != null) return _name_xjal;

_name_xjal = checkNameOfShape_xjal( _shape, text14, "text14" ); if (_name_xjal != null) return _name_xjal;

_name_xjal = checkNameOfShape_xjal( _shape, line10, "line10" ); if (_name_xjal != null) return _name_xjal;

_name_xjal = checkNameOfShape_xjal( _shape, text15, "text15" ); if (_name_xjal != null) return _name_xjal;

_name_xjal = checkNameOfShape_xjal( _shape, line11, "line11" ); if (_name_xjal != null) return _name_xjal;

_name_xjal = checkNameOfShape_xjal( _shape, text5, "text5" ); if (_name_xjal != null) return _name_xjal;

_name_xjal = checkNameOfShape_xjal( _shape, line2, "line2" ); if (_name_xjal != null) return _name_xjal;

_name_xjal = checkNameOfShape_xjal( _shape, text4, "text4" ); if (_name_xjal != null) return _name_xjal;

_name_xjal = checkNameOfShape_xjal( _shape, line3, "line3" ); if (_name_xjal != null) return _name_xjal;

_name_xjal = checkNameOfShape_xjal( _shape, text7, "text7" ); if (_name_xjal != null) return _name_xjal;

_name_xjal = checkNameOfShape_xjal( _shape, text13, "text13" ); if (_name_xjal != null) return _name_xjal;

_name_xjal = checkNameOfShape_xjal( _shape, line9, "line9" ); if (_name_xjal != null) return _name_xjal;

} catch (Exception e) {

return null;

}

return super.getNameOfShape_xjal( _shape );

}

@AnyLogicInternalCodegenAPI

protected static final int[] _connector6_pointsX_xjal = {

530, 580 };

@AnyLogicInternalCodegenAPI

protected static final int[] _connector6_pointsY_xjal = {

180,180 };

@AnyLogicInternalCodegenAPI

protected static final int[] _connector1_pointsX_xjal = {

660, 620 };

@AnyLogicInternalCodegenAPI

protected static final int[] _connector1_pointsY_xjal = {

180,180 };

@AnyLogicInternalCodegenAPI

protected static final int[] _connector_pointsX_xjal = {

30, 30, 0, 0, 0, 40, 40, 40 };

@AnyLogicInternalCodegenAPI

protected static final int[] _connector_pointsY_xjal = {

530,400, 400, 340, 250, 250, 220, 180 };

@AnyLogicInternalCodegenAPI

protected static final int[] _connector10_pointsX_xjal = {

320, 320, 280, 280, 300, 300 };

@AnyLogicInternalCodegenAPI

protected static final int[] _connector10_pointsY_xjal = {

180,280, 280, 390, 390, 530 };

@AnyLogicInternalCodegenAPI

protected static final int[] _connector7_pointsX_xjal = {

520, 520, 470, 470, 510, 510 };

@AnyLogicInternalCodegenAPI

protected static final int[] _connector7_pointsY_xjal = {

190,230, 230, 300, 300, 400 };

@AnyLogicInternalCodegenAPI

protected static final int[] _connector14_pointsX_xjal = {

370, 360 };

@AnyLogicInternalCodegenAPI

protected static final int[] _connector14_pointsY_xjal = {

180,180 };

@AnyLogicInternalCodegenAPI

protected static final int[] _connector15_pointsX_xjal = {

100, 80 };

@AnyLogicInternalCodegenAPI

protected static final int[] _connector15_pointsY_xjal = {

180,180 };

@AnyLogicInternalCodegenAPI

protected static final int[] _connector11_pointsX_xjal = {

510, 440 };

@AnyLogicInternalCodegenAPI

protected static final int[] _connector11_pointsY_xjal = {

180,180 };

@AnyLogicInternalCodegenAPI

protected static final int[] _connector13_pointsX_xjal = {

400, 390 };

@AnyLogicInternalCodegenAPI

protected static final int[] _connector13_pointsY_xjal = {

180,180 };

@AnyLogicInternalCodegenAPI

protected static final int[] _connector8_pointsX_xjal = {

220, 170 };

@AnyLogicInternalCodegenAPI

protected static final int[] _connector8_pointsY_xjal = {

180,180 };

@AnyLogicInternalCodegenAPI

protected static final int[] _connector16_pointsX_xjal = {

130, 120 };

@AnyLogicInternalCodegenAPI

protected static final int[] _connector16_pointsY_xjal = {

180,180 };

@AnyLogicInternalCodegenAPI

protected static final int[] _connector2_pointsX_xjal = {

240, 320 };

@AnyLogicInternalCodegenAPI

protected static final int[] _connector2_pointsY_xjal = {

180,180 };

@AnyLogicInternalCodegenAPI

protected static final int[] _connector3_pointsX_xjal = {

230, 210 };

@AnyLogicInternalCodegenAPI

protected static final int[] _connector3_pointsY_xjal = {

190,280 };

@AnyLogicInternalCodegenAPI

protected static final int[] _connector4_pointsX_xjal = {

250, 230 };

@AnyLogicInternalCodegenAPI

protected static final int[] _connector4_pointsY_xjal = {

280,400 };

@AnyLogicInternalCodegenAPI

private void drawModelElements_Events_xjal(Panel _panel, Graphics2D _g, boolean _publicOnly, boolean _isSuperClass ) {

if (!_publicOnly) {

drawEvent( _panel, _g, 200, -50, 10, 0, "event", event );

}

if (!_publicOnly) {

drawEvent( _panel, _g, 310, -50, 10, 0, "event1", event1 );

}

if (!_publicOnly) {

drawEvent( _panel, _g, 430, -50, 10, 0, "event2", event2 );

}

if (!_publicOnly) {

drawEvent( _panel, _g, 510, -50, 10, 0, "event3", event3 );

}

}

@AnyLogicInternalCodegenAPI

private void drawModelElements_Parameters_xjal(Panel _panel, Graphics2D _g, boolean _publicOnly, boolean _isSuperClass ) {

if (!_publicOnly) {

drawParameter( _panel, _g, 310, 970, 10, 0, "p1", p1, 0 );

}

if (!_publicOnly) {

drawParameter( _panel, _g, 410, 990, 10, 0, "u", u, 0 );

}

if (!_publicOnly) {

drawParameter( _panel, _g, 470, 990, 10, 0, "x", x, 0 );

}

if (!_publicOnly) {

drawParameter( _panel, _g, 560, 990, 10, 0, "l", l, 0 );

}

}

@AnyLogicInternalCodegenAPI

private void drawModelElements_EmbeddeObjects_xjal(Panel _panel, Graphics2D _g, boolean _publicOnly, boolean _isSuperClass ) {

// Embedded object "Supplier0"

if (!_publicOnly) {

drawEmbeddedObjectModel( _panel, _g, -20 , 130 , 35, 15, "Supplier0", this.Supplier0 );

}

// Embedded object "Distributor0"

if (!_publicOnly) {

drawEmbeddedObjectModel( _panel, _g, 260 , 130 , 40, 0, "Distributor0", this.Distributor0 );

}

// Embedded object "Retailer0"

if (!_publicOnly) {

drawEmbeddedObjectModel( _panel, _g, 520 , 130 , 55, 15, "Retailer0", this.Retailer0 );

}

// Embedded object "selectOutput"

if (!_publicOnly) {

drawEmbeddedObjectModel( _panel, _g, 160 , 130 , 15, 15, "selectOutput", this.selectOutput );

}

// Embedded object "sink"

if (!_publicOnly) {

drawEmbeddedObjectModel( _panel, _g, 180 , 350 , 45, 85, "sink", this.sink );

}

// Embedded object "selectOutput1"

if (!_publicOnly) {

drawEmbeddedObjectModel( _panel, _g, 450 , 130 , 25, 20, "selectOutput1", this.selectOutput1 );

}

// Embedded object "sink1"

if (!_publicOnly) {

drawEmbeddedObjectModel( _panel, _g, 460 , 350 , 45, 85, "sink1", this.sink1 );

}

// Embedded object "sink2"

if (!_publicOnly) {

drawEmbeddedObjectModel( _panel, _g, 610 , 130 , 40, 20, "sink2", this.sink2 );

}

// Embedded object "Manufacturer0"

if (!_publicOnly) {

drawEmbeddedObjectModel( _panel, _g, -20 , 480 , 65, 55, "Manufacturer0", this.Manufacturer0 );

}

// Embedded object "Manufacturer1"

if (!_publicOnly) {

drawEmbeddedObjectModel( _panel, _g, 250 , 480 , 65, 45, "Manufacturer1", this.Manufacturer1 );

}

// Embedded object "hold"

if (!_publicOnly) {

drawEmbeddedObjectModel( _panel, _g, 50 , 130 , 45, 20, "hold", this.hold );

}

// Embedded object "hold1"

if (!_publicOnly) {

drawEmbeddedObjectModel( _panel, _g, 320 , 130 , 45, 20, "hold1", this.hold1 );

}

// Embedded object "delay1"

if (!_publicOnly) {

drawEmbeddedObjectModel( _panel, _g, 340 , 130 , 60, 15, "delay1", this.delay1 );

}

// Embedded object "delay"

if (!_publicOnly) {

drawEmbeddedObjectModel( _panel, _g, 70 , 130 , 60, 15, "delay", this.delay );

}

// Embedded object "delay2"

if (!_publicOnly) {

drawEmbeddedObjectModel( _panel, _g, 150 , 230 , 60, 15, "delay2", this.delay2 );

}

}

@AnyLogicInternalCodegenAPI

private void drawModelElements_Connectors_xjal(Panel _panel, Graphics2D _g, boolean _publicOnly, boolean _isSuperClass ) {

if (!_publicOnly) {

drawConnector( _panel, _g, _connector6_pointsX_xjal, _connector6_pointsY_xjal, false );

}

if (!_publicOnly) {

drawConnector( _panel, _g, _connector1_pointsX_xjal, _connector1_pointsY_xjal, false );

}

if (!_publicOnly) {

drawConnector( _panel, _g, _connector_pointsX_xjal, _connector_pointsY_xjal, false );

}

if (!_publicOnly) {

drawConnector( _panel, _g, _connector10_pointsX_xjal, _connector10_pointsY_xjal, false );

}

if (!_publicOnly) {

drawConnector( _panel, _g, _connector7_pointsX_xjal, _connector7_pointsY_xjal, false );

}

if (!_publicOnly) {

drawConnector( _panel, _g, _connector14_pointsX_xjal, _connector14_pointsY_xjal, false );

}

if (!_publicOnly) {

drawConnector( _panel, _g, _connector15_pointsX_xjal, _connector15_pointsY_xjal, false );

}

if (!_publicOnly) {

drawConnector( _panel, _g, _connector11_pointsX_xjal, _connector11_pointsY_xjal, false );

}

if (!_publicOnly) {

drawConnector( _panel, _g, _connector13_pointsX_xjal, _connector13_pointsY_xjal, false );

}

if (!_publicOnly) {

drawConnector( _panel, _g, _connector8_pointsX_xjal, _connector8_pointsY_xjal, false );

}

if (!_publicOnly) {

drawConnector( _panel, _g, _connector16_pointsX_xjal, _connector16_pointsY_xjal, false );

}

if (!_publicOnly) {

drawConnector( _panel, _g, _connector2_pointsX_xjal, _connector2_pointsY_xjal, false );

}

if (!_publicOnly) {

drawConnector( _panel, _g, _connector3_pointsX_xjal, _connector3_pointsY_xjal, false );

}

if (!_publicOnly) {

drawConnector( _panel, _g, _connector4_pointsX_xjal, _connector4_pointsY_xjal, false );

}

}

@AnyLogicInternalCodegenAPI

private void drawModelElements_AgentLinks_xjal(Panel _panel, Graphics2D _g, boolean _publicOnly, boolean _isSuperClass ) {

if (_publicOnly) { return; }

drawLinkToAgent( _panel, _g, 50, -50, 15, 0, "connections", true, connections );

}

@Override

@AnyLogicInternalCodegenAPI

public void drawModelElements( Panel _panel, Graphics2D _g, boolean _publicOnly, boolean _isSuperClass ) {

super.drawModelElements( _panel, _g, _publicOnly, true );

drawModelElements_Events_xjal( _panel, _g, _publicOnly, _isSuperClass );

drawModelElements_Parameters_xjal( _panel, _g, _publicOnly, _isSuperClass );

drawModelElements_EmbeddeObjects_xjal( _panel, _g, _publicOnly, _isSuperClass );

drawModelElements_Connectors_xjal( _panel, _g, _publicOnly, _isSuperClass );

drawModelElements_AgentLinks_xjal( _panel, _g, _publicOnly, _isSuperClass );

}

@AnyLogicInternalCodegenAPI

private boolean onClickModelAt_EmbeddedObjects_xjal( Panel _panel, double _x, double _y, int _clickCount, boolean _publicOnly, boolean _isSuperClass ) {

if ( Supplier0.onClickIconAt( _x - -20, _y - 130, true ) ) {

if ( _clickCount == 2 ) {

_panel.browseAgent_xjal( _x, _y, this, "Supplier0" );

} else {

_panel.addInspect( _x, _y, this, "Supplier0" );

}

return true;

}

if ( Distributor0.onClickIconAt( _x - 260, _y - 130, true ) ) {

if ( _clickCount == 2 ) {

_panel.browseAgent_xjal( _x, _y, this, "Distributor0" );

} else {

_panel.addInspect( _x, _y, this, "Distributor0" );

}

return true;

}

if ( Retailer0.onClickIconAt( _x - 520, _y - 130, true ) ) {

if ( _clickCount == 2 ) {

_panel.browseAgent_xjal( _x, _y, this, "Retailer0" );

} else {

_panel.addInspect( _x, _y, this, "Retailer0" );

}

return true;

}

if ( selectOutput.onClickIconAt( _x - 160, _y - 130, true ) ) {

if ( _clickCount == 2 ) {

_panel.browseAgent_xjal( _x, _y, this, "selectOutput" );

} else {

_panel.addInspect( _x, _y, this, "selectOutput" );

}

return true;

}

if ( sink.onClickIconAt( _x - 180, _y - 350, true ) ) {

if ( _clickCount == 2 ) {

_panel.browseAgent_xjal( _x, _y, this, "sink" );

} else {

_panel.addInspect( _x, _y, this, "sink" );

}

return true;

}

if ( selectOutput1.onClickIconAt( _x - 450, _y - 130, true ) ) {

if ( _clickCount == 2 ) {

_panel.browseAgent_xjal( _x, _y, this, "selectOutput1" );

} else {

_panel.addInspect( _x, _y, this, "selectOutput1" );

}

return true;

}

if ( sink1.onClickIconAt( _x - 460, _y - 350, true ) ) {

if ( _clickCount == 2 ) {

_panel.browseAgent_xjal( _x, _y, this, "sink1" );

} else {

_panel.addInspect( _x, _y, this, "sink1" );

}

return true;

}

if ( sink2.onClickIconAt( _x - 610, _y - 130, true ) ) {

if ( _clickCount == 2 ) {

_panel.browseAgent_xjal( _x, _y, this, "sink2" );

} else {

_panel.addInspect( _x, _y, this, "sink2" );

}

return true;

}

if ( Manufacturer0.onClickIconAt( _x - -20, _y - 480, true ) ) {

if ( _clickCount == 2 ) {

_panel.browseAgent_xjal( _x, _y, this, "Manufacturer0" );

} else {

_panel.addInspect( _x, _y, this, "Manufacturer0" );

}

return true;

}

if ( Manufacturer1.onClickIconAt( _x - 250, _y - 480, true ) ) {

if ( _clickCount == 2 ) {

_panel.browseAgent_xjal( _x, _y, this, "Manufacturer1" );

} else {

_panel.addInspect( _x, _y, this, "Manufacturer1" );

}

return true;

}

if ( hold.onClickIconAt( _x - 50, _y - 130, true ) ) {

if ( _clickCount == 2 ) {

_panel.browseAgent_xjal( _x, _y, this, "hold" );

} else {

_panel.addInspect( _x, _y, this, "hold" );

}

return true;

}

if ( hold1.onClickIconAt( _x - 320, _y - 130, true ) ) {

if ( _clickCount == 2 ) {

_panel.browseAgent_xjal( _x, _y, this, "hold1" );

} else {

_panel.addInspect( _x, _y, this, "hold1" );

}

return true;

}

if ( delay1.onClickIconAt( _x - 340, _y - 130, true ) ) {

if ( _clickCount == 2 ) {

_panel.browseAgent_xjal( _x, _y, this, "delay1" );

} else {

_panel.addInspect( _x, _y, this, "delay1" );

}

return true;

}

if ( delay.onClickIconAt( _x - 70, _y - 130, true ) ) {

if ( _clickCount == 2 ) {

_panel.browseAgent_xjal( _x, _y, this, "delay" );

} else {

_panel.addInspect( _x, _y, this, "delay" );

}

return true;

}

if ( delay2.onClickIconAt( _x - 150, _y - 230, true ) ) {

if ( _clickCount == 2 ) {

_panel.browseAgent_xjal( _x, _y, this, "delay2" );

} else {

_panel.addInspect( _x, _y, this, "delay2" );

}

return true;

}

return false;

}

@AnyLogicInternalCodegenAPI

private boolean onClickModelAt_AgentLinks_xjal( Panel _panel, double _x, double _y, int _clickCount, boolean _publicOnly, boolean _isSuperClass ) {

if ( modelElementContains(_x, _y, 50, -50) ) {

_panel.addInspect_xjal( 50, -50, this, "connections", Panel.INSPECT_CONNECTIONS_xjal );

return true;

}

return false;

}

@AnyLogicInternalCodegenAPI

private boolean onClickModelAt_Parameters_xjal( Panel _panel, double _x, double _y, int _clickCount, boolean _publicOnly, boolean _isSuperClass ) {

if( !_publicOnly && modelElementContains(_x, _y, 310, 970) ) {

_panel.addInspect( 310, 970, this, "p1" );

return true;

}

if( !_publicOnly && modelElementContains(_x, _y, 410, 990) ) {

_panel.addInspect( 410, 990, this, "u" );

return true;

}

if( !_publicOnly && modelElementContains(_x, _y, 470, 990) ) {

_panel.addInspect( 470, 990, this, "x" );

return true;

}

if( !_publicOnly && modelElementContains(_x, _y, 560, 990) ) {

_panel.addInspect( 560, 990, this, "l" );

return true;

}

return false;

}

@AnyLogicInternalCodegenAPI

private boolean onClickModelAt_Events_xjal( Panel _panel, double _x, double _y, int _clickCount, boolean _publicOnly, boolean _isSuperClass ) {

if( !_publicOnly && modelElementContains(_x, _y, 200, -50) ) {

_panel.addInspect( 200, -50, this, "event" );

return true;

}

if( !_publicOnly && modelElementContains(_x, _y, 310, -50) ) {

_panel.addInspect( 310, -50, this, "event1" );

return true;

}

if( !_publicOnly && modelElementContains(_x, _y, 430, -50) ) {

_panel.addInspect( 430, -50, this, "event2" );

return true;

}

if( !_publicOnly && modelElementContains(_x, _y, 510, -50) ) {

_panel.addInspect( 510, -50, this, "event3" );

return true;

}

return false;

}

@Override

@AnyLogicInternalCodegenAPI

public boolean onClickModelAt( Panel _panel, double _x, double _y, int _clickCount, boolean _publicOnly, boolean _isSuperClass ) {

if ( onClickModelAt_EmbeddedObjects_xjal( _panel, _x, _y, _clickCount, _publicOnly, _isSuperClass ) ) { return true; }

if ( onClickModelAt_AgentLinks_xjal( _panel, _x, _y, _clickCount, _publicOnly, _isSuperClass ) ) { return true; }

if ( onClickModelAt_Parameters_xjal( _panel, _x, _y, _clickCount, _publicOnly, _isSuperClass ) ) { return true; }

if ( onClickModelAt_Events_xjal( _panel, _x, _y, _clickCount, _publicOnly, _isSuperClass ) ) { return true; }

return super.onClickModelAt( _panel, _x, _y, _clickCount, _publicOnly, true );

}

/**

* 构造器

*/

public Main( Engine engine, Agent owner, AgentList<? extends Main> ownerPopulation ) {

super( engine, owner, ownerPopulation );

instantiateBaseStructureThis_xjal();

}

@AnyLogicInternalCodegenAPI

public void onOwnerChanged_xjal() {

super.onOwnerChanged_xjal();

setupReferences_xjal();

}

@AnyLogicInternalCodegenAPI

public void instantiateBaseStructure_xjal() {

super.instantiateBaseStructure_xjal();

instantiateBaseStructureThis_xjal();

}

@AnyLogicInternalCodegenAPI

private void instantiateBaseStructureThis_xjal() {

Supplier0 = instantiate_Supplier0_xjal();

Distributor0 = instantiate_Distributor0_xjal();

Retailer0 = instantiate_Retailer0_xjal();

selectOutput = instantiate_selectOutput_xjal();

sink = instantiate_sink_xjal();

selectOutput1 = instantiate_selectOutput1_xjal();

sink1 = instantiate_sink1_xjal();

sink2 = instantiate_sink2_xjal();

Manufacturer0 = instantiate_Manufacturer0_xjal();

Manufacturer1 = instantiate_Manufacturer1_xjal();

hold = instantiate_hold_xjal();

hold1 = instantiate_hold1_xjal();

delay1 = instantiate_delay1_xjal();

delay = instantiate_delay_xjal();

delay2 = instantiate_delay2_xjal();

setupReferences_xjal();

}

@AnyLogicInternalCodegenAPI

private void setupReferences_xjal() {

}

/**

* Simple constructor. Please add created agent to some population by calling goToPopulation() function

*/

public Main() {

}

/**

* Simple constructor. Please add created agent to some population by calling goToPopulation() function

*/

public Main( double p1, double u, double x, double l ) {

markParametersAreSet();

this.p1 = p1;

this.u = u;

this.x = x;

this.l = l;

}

/**

* 创建嵌入对象实例

*/

@AnyLogicInternalCodegenAPI

private void instantiatePopulations_xjal() {

}

@Override

@AnyLogicInternalCodegenAPI

public void doCreate() {

super.doCreate();

// 创建嵌入对象实例

instantiatePopulations_xjal();

// 为简单变量赋初始值

setupPlainVariables_Main_xjal();

// 持久元素的动态初始化

_createPersistentElementsAP0_xjal();

presentation = new ShapeTopLevelPresentationGroup( Main.this, true, 0, 0, 0, 0 , text, text1, line, text2, line1, text3, text8, line4, text6, line5, text9, line6, text10, line7, text11, line8, text12, text14, line10, text15, line11, text5, line2, text4, line3, text7, text13, line9, slider2, slider, plot, plot1, plot3, plot2, plot4 );

icon = new ShapeGroup( Main.this, true, 0, 0, 0 );

// 创建嵌入对象实例

instantiatePopulations_xjal();

// 创建非重复嵌入对象

setupParameters_Supplier0_xjal( Supplier0 );

create_Supplier0_xjal( Supplier0 );

setupParameters_Distributor0_xjal( Distributor0 );

create_Distributor0_xjal( Distributor0 );

setupParameters_Retailer0_xjal( Retailer0 );

create_Retailer0_xjal( Retailer0 );

setupParameters_selectOutput_xjal( selectOutput );

create_selectOutput_xjal( selectOutput );

setupParameters_sink_xjal( sink );

create_sink_xjal( sink );

setupParameters_selectOutput1_xjal( selectOutput1 );

create_selectOutput1_xjal( selectOutput1 );

setupParameters_sink1_xjal( sink1 );

create_sink1_xjal( sink1 );

setupParameters_sink2_xjal( sink2 );

create_sink2_xjal( sink2 );

setupParameters_Manufacturer0_xjal( Manufacturer0 );

create_Manufacturer0_xjal( Manufacturer0 );

setupParameters_Manufacturer1_xjal( Manufacturer1 );

create_Manufacturer1_xjal( Manufacturer1 );

setupParameters_hold_xjal( hold );

create_hold_xjal( hold );

setupParameters_hold1_xjal( hold1 );

create_hold1_xjal( hold1 );

setupParameters_delay1_xjal( delay1 );

create_delay1_xjal( delay1 );

setupParameters_delay_xjal( delay );

create_delay_xjal( delay );

setupParameters_delay2_xjal( delay2 );

create_delay2_xjal( delay2 );

// 非重复对象的端口连接器

selectOutput1.outT.connect( Retailer0.in ); // connector6

sink2.in.connect( Retailer0.out ); // connector1

Manufacturer0.out.connect( Supplier0.in ); // connector

Distributor0.in.connect( Manufacturer1.out ); // connector10

selectOutput1.outF.connect( sink1.in ); // connector7

hold1.in.connect( Distributor0.out ); // connector14

hold.in.connect( Supplier0.out ); // connector15

selectOutput1.in.connect( delay1.out ); // connector11

delay1.in.connect( hold1.out ); // connector13

selectOutput.in.connect( delay.out ); // connector8

delay.in.connect( hold.out ); // connector16

selectOutput.outT.connect( Distributor0.in ); // connector2

selectOutput.outF.connect( delay2.in ); // connector3

delay2.out.connect( sink.in ); // connector4

// 创建重复嵌入对象

setupInitialConditions_xjal( Main.class );

slider2.setValueToDefault();

slider.setValueToDefault();

}

@AnyLogicInternalCodegenAPI

public void setupExt_xjal(AgentExtension _ext) {

// 智能体属性设置

if ( _ext instanceof ExtAgentWithSpatialMetrics && _ext instanceof ExtWithSpaceType ) {

double _value;

_value =

1

;

((ExtAgentWithSpatialMetrics) _ext).setSpeed( _value, MPS );

}

}

@Override

@AnyLogicInternalCodegenAPI

public void doStart() {

super.doStart();

event.start();

event1.start();

event2.start();

event3.start();

_plot_autoUpdateEvent_xjal.start();

_plot1_autoUpdateEvent_xjal.start();

_plot3_autoUpdateEvent_xjal.start();

_plot2_autoUpdateEvent_xjal.start();

_plot4_autoUpdateEvent_xjal.start();

Supplier0.start();

Distributor0.start();

Retailer0.start();

selectOutput.start();

sink.start();

selectOutput1.start();

sink1.start();

sink2.start();

Manufacturer0.start();

Manufacturer1.start();

hold.start();

hold1.start();

delay1.start();

delay.start();

delay2.start();

}

@AnyLogicInternalCodegenAPI

public void onStartup() {

super.onStartup();

Manufacturer0.inject(50);

Manufacturer1.inject(50);

}

/**

* 为简单变量赋初始值<br>

* <em>This method isn't designed to be called by user and may be removed in future releases.</em>

*/

@AnyLogicInternalCodegenAPI

public void setupPlainVariables_xjal() {

setupPlainVariables_Main_xjal();

}

/**

* 为简单变量赋初始值<br>

* <em>This method isn't designed to be called by user and may be removed in future releases.</em>

*/

@AnyLogicInternalCodegenAPI

private void setupPlainVariables_Main_xjal() {

}

// 用户API -----------------------------------------------------

@AnyLogicInternalCodegenAPI

static LinkToAgentAnimationSettings _connections_commonAnimationSettings_xjal = new LinkToAgentAnimationSettingsImpl( false, black, 1.0, LINE_STYLE_SOLID, ARROW_NONE, 0.0 );

public LinkToAgentCollection<Agent, Agent> connections = new LinkToAgentStandardImpl<Agent, Agent>(this, _connections_commonAnimationSettings_xjal);

@Override

public LinkToAgentCollection<? extends Agent, ? extends Agent> getLinkToAgentStandard_xjal() {

return connections;

}

@AnyLogicInternalCodegenAPI

public void drawLinksToAgents(boolean _underAgents_xjal, LinkToAgentAnimator _animator_xjal) {

super.drawLinksToAgents(_underAgents_xjal, _animator_xjal);

if ( _underAgents_xjal ) {

_animator_xjal.drawLink( this, connections, true, true );

}

}

public List<Object> getEmbeddedObjects() {

List<Object> list = super.getEmbeddedObjects();

if (list == null) {

list = new LinkedList<Object>();

}

list.add( Supplier0 );

list.add( Distributor0 );

list.add( Retailer0 );

list.add( selectOutput );

list.add( sink );

list.add( selectOutput1 );

list.add( sink1 );

list.add( sink2 );

list.add( Manufacturer0 );

list.add( Manufacturer1 );

list.add( hold );

list.add( hold1 );

list.add( delay1 );

list.add( delay );

list.add( delay2 );

return list;

}

public AgentList<? extends Main> getPopulation() {

return (AgentList<? extends Main>) super.getPopulation();

}

public List<? extends Main> agentsInRange( double distance ) {

return (List<? extends Main>) super.agentsInRange( distance );

}

@Override

@AnyLogicInternalCodegenAPI

public boolean isLoggingToDB(EventOriginator _e) {

if ( _e == _plot_autoUpdateEvent_xjal ) return false;

if ( _e == _plot1_autoUpdateEvent_xjal ) return false;

if ( _e == _plot3_autoUpdateEvent_xjal ) return false;

if ( _e == _plot2_autoUpdateEvent_xjal ) return false;

if ( _e == _plot4_autoUpdateEvent_xjal ) return false;

return super.isLoggingToDB( _e );

}

@AnyLogicInternalCodegenAPI

public void onDestroy() {

event.onDestroy();

event1.onDestroy();

event2.onDestroy();

event3.onDestroy();

_plot_autoUpdateEvent_xjal.onDestroy();

_plot1_autoUpdateEvent_xjal.onDestroy();

_plot3_autoUpdateEvent_xjal.onDestroy();

_plot2_autoUpdateEvent_xjal.onDestroy();

_plot4_autoUpdateEvent_xjal.onDestroy();

Supplier0.onDestroy();

Distributor0.onDestroy();

Retailer0.onDestroy();

selectOutput.onDestroy();

sink.onDestroy();

selectOutput1.onDestroy();

sink1.onDestroy();

sink2.onDestroy();

Manufacturer0.onDestroy();

Manufacturer1.onDestroy();

hold.onDestroy();

hold1.onDestroy();

delay1.onDestroy();

delay.onDestroy();

delay2.onDestroy();

_plot_expression0_dataSet_xjal.destroyUpdater_xjal();

_plot1_expression0_dataSet_xjal.destroyUpdater_xjal();

_plot3_expression0_dataSet_xjal.destroyUpdater_xjal();

_plot2_expression0_dataSet_xjal.destroyUpdater_xjal();

_plot4_expression0_dataSet_xjal.destroyUpdater_xjal();

logToDB( _plot_expression0_dataSet_xjal, "plot : Quantity of Supplier" );

logToDB( _plot1_expression0_dataSet_xjal, "plot1 : Quantity of Distributor" );

logToDB( _plot3_expression0_dataSet_xjal, "plot3 : Dataset Title" );

logToDB( _plot2_expression0_dataSet_xjal, "plot2 : Dataset Title" );

logToDB( _plot4_expression0_dataSet_xjal, "plot4 : Dataset Title" );

super.onDestroy();

}

/**

* 这个数字在这里是出于模型快照存储的目的。它不应该被用户修改。

*/

@AnyLogicInternalCodegenAPI

private static final long serialVersionUID = -6773233646951151800L;

}
